# Graph-based multi-modality integration for prediction of cancer subtype and severity

**DOI:** 10.1038/s41598-023-46392-6

**Published:** 2023-11-10

**Authors:** Diane Duroux, Christian Wohlfart, Kristel Van Steen, Antoaneta Vladimirova, Michael King

**Affiliations:** 1 BIO3 - Systems Genetics, GIGA-R Medical Genomics, University of Liège, 4000 Liège, Belgium; 2 Roche Diagnostics GmbH, Penzberg, Germany; 3 Department of Human Genetics, BIO3 - Systems Medicine, 3000 Leuven, Belgium; 4 Roche Information Solutions, Roche Diagnostics Corporation, Santa Clara, California, United States of America; 5 Post-Doctoral Fellow, ETH AI center, Zürich, Switzerland

**Keywords:** Computational biology and bioinformatics, Systems biology

## Abstract

Personalised cancer screening before therapy paves the way toward improving diagnostic accuracy and treatment outcomes. Most approaches are limited to a single data type and do not consider interactions between features, leaving aside the complementary insights that multimodality and systems biology can provide. In this project, we demonstrate the use of graph theory for data integration via individual networks where nodes and edges are individual-specific. We showcase the consequences of early, intermediate, and late graph-based fusion of RNA-Seq data and histopathology whole-slide images for predicting cancer subtypes and severity. The methodology developed is as follows: (1) we create individual networks; (2) we compute the similarity between individuals from these graphs; (3) we train our model on the similarity matrices; (4) we evaluate the performance using the macro F1 score. Pros and cons of elements of the pipeline are evaluated on publicly available real-life datasets. We find that graph-based methods can increase performance over methods that do not study interactions. Additionally, merging multiple data sources often improves classification compared to models based on single data, especially through intermediate fusion. The proposed workflow can easily be adapted to other disease contexts to accelerate and enhance personalized healthcare.

## Introduction

Disease subtyping refers to the identification of homogeneous groups of patients. It can be used to detect a disease’s severity or target treatments with the highest probability of success. Disease subtyping is essential in cancer research^9,51^ since cancers are highly diverse and severe. Many methods for disease subtyping analyses rely on a single data modality only. However, one modality is unlikely to be informative enough to capture the whole complexity of complex diseases. In addition, a large panel of data is available, making multi-modality integration realistic. More and more studies investigate combining multiple data sources^26,36^ to overcome the oversimplification of approaches based on a unique data type. For instance, multiple studies investigate the benefits of combining images, and genomic data^1,22,44^.

Often, late integration is performed. In such studies, the data sources are investigated independently before classification results are merged. Nevertheless, independent analyses may lead to inconsistent outcomes that are complex to aggregate. The main disadvantages of this method are that it does not take advantage of the possible complementarity of the modalities. Another standard integration procedure is early fusion, which considers the concatenation of the data sources before applying a machine-learning model. Whereas this solution is simple to implement, concatenation may decrease the signal-to-noise ratio in each data modality if not processed appropriately, as it increases the input space’s dimensionality, and potentially introduces irrelevant information that can decrease the model’s performance. In the last decade, several developments have been proposed to combine data between the start and end steps to solve these issues. For example, iCluster^47,48^ performs data fusion and dimensionality reduction at the same time. This method uses a Gaussian latent variable model with lasso-type penalty terms to induce sparsity in the coefficient matrices toward feature selection. One drawback of this approach is its high computational complexity. An alternative is Affinity Aggregation for Spectral Clustering^23^. The main idea is to compute a matrix of similarity between samples for each data source (Supplementary Figure 1). Then, these multiple affinity networks are clustered via Spectral Clustering using linear combination with weights optimised using multiple kernel learning. In the same vein, Similarity Network Fusion (SNF) (^53^) was implemented to combine multiple similarity matrices into a single one by iteratively updating the matrices to make them more and more similar until the algorithm converges. This final matrix becomes the new input to the classification algorithm. Later, regularised unsupervised multiple kernel learning^49^ was introduced. It extends multiple kernel learning for dimensionality reduction approaches with a regularisation term to avoid overfitting during the optimisation procedure and using several kernels per data type.

Most of the graph analyses for complex diseases aggregate information across a whole cohort, failing to detect individual characteristics^16^. Exploiting individual-specific interactions rather than population-level systems will help capture the heterogeneity between individuals and enhance the identification of new biomarkers for precision medicine. This observation paves the way for developing individual networks (INs). INs are networks where nodes and/or edges are individual-specific. For each individual, nodes are variables (e.g., genes), and edges show the link between these variables for that individual. Since INs represent individual relations between variables, we can readily use them for precision medicine. Individual networks can be inferred via multiple approaches. For example, individual’s variables values (e.g., gene expression) can be superimposed to a reference network obtained from external knowledge (e.g., protein interactions)^39^, but then only node values will differ between individuals and not the graph topology. Another option is Linear Interpolation to Obtain Network Estimates for Single Samples (LIONESS)^32^. *LIONESS* computes edge weights from the difference in edge weights for a network constructed using all the samples and a network reconstructed using all but the sample of interest. Single sample networks based on the Pearson correlation (ssPCC) algorithm^37^ are also built from the perturbation of an individual against a group of given samples. These individual networks are derived from the perturbation of the Pearson correlation caused by the addition of this individual from a different population. Alternatively, an edge weight can be computed without a reference panel by adding Z-scores of log-transformed values of the two associated nodes^29^ or by using repeated measurements per variable per individual. Recent work^11^ explores individual networks for clustering tasks, which fall under unsupervised learning. In contrast, this work focuses on the supervised scenario, where we harness the power of INs for supervised tasks. The performance of supervised data integration methods in association with INs remains to be investigated. In this project, we explore three main questions. We study to what extent it can predict disease subtypes and severity from the patient data. We examine which modality yields the best prediction. We leverage the consequences of using individual networks to combine two modalities at various steps, keeping the rest of the pipeline unchanged.

## Materials and methods

**Figure 1:**
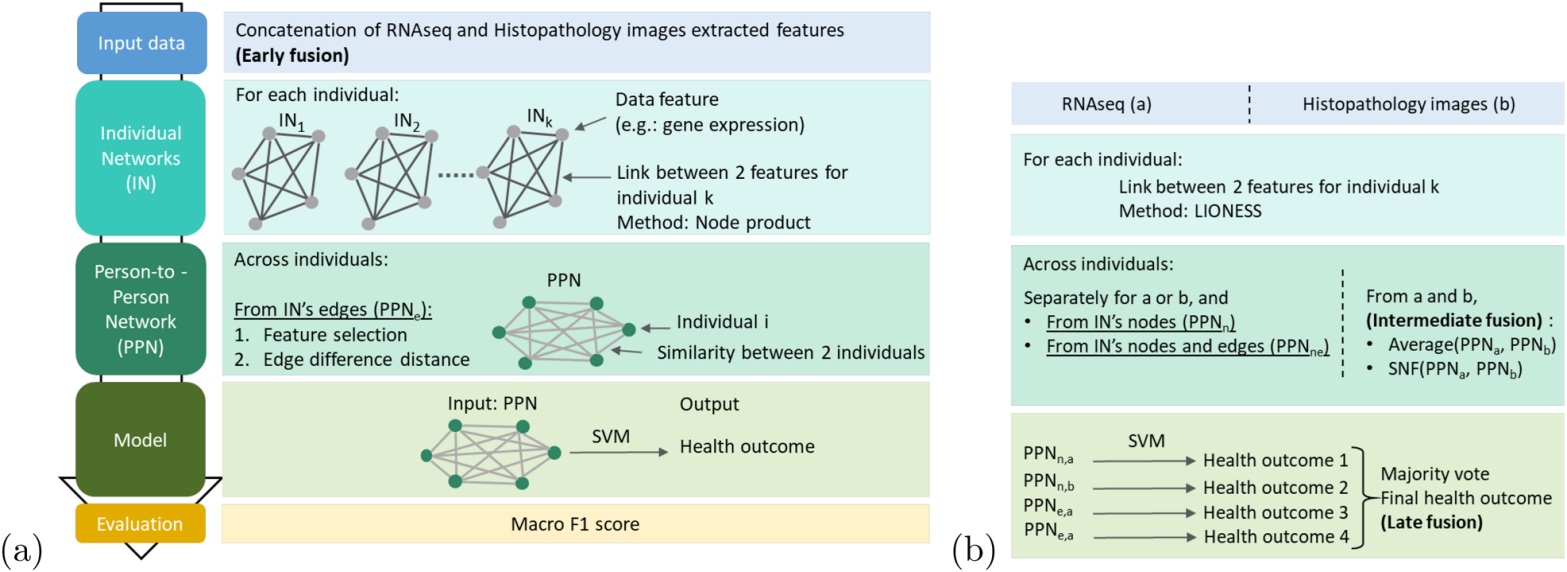
(**a**) Data-integration workflow. The input is the concatenation of the RNA-Seq data, and the histopathology images extracted features (Sect. “Data”). An Individual Network (IN) is constructed for each individual of the train set. From these INs, a Person-to-Person Network (PPN) is built, where nodes are individuals and edges represent how close two individuals are. Only the edges from the individual graph are used to build the PPN. The PPN is used to train an SVM model. Then, the individual networks of the test set are computed. The similarities of the individuals from the test set to the individuals from the train set are calculated to create the PPN of the test set. The SVM model is applied to these similarities to a reference set, and the performance is determined using the macro F1 score. (**b**) Variations of the workflow. Two additional inputs are considered: the RNA-Seq modality only, and the histopathology images only. The edges of the INs are constructed from the *LIONESS* methods. To build the PPN, either the nodes only or the nodes and the edges from the INs are used. The variations and the combinations of variations (summarized in supplementary Fig. 2) are compared.

We developed a multi-step workflow (see Fig. 1) to predict outcomes via individual graphs. First, a network is constructed for each individual: nodes and/or edges are specific to an individual. From these individual networks, we compute a similarity matrix that we call a Person-to-Person Network (PPN): nodes are individuals and edges represent how similar individuals are. Various levels of information from the individual graph are used to build the Person-to-Person network: nodes, edges, or nodes and edges. The Person-to-Person network becomes the input of the machine learning model. In other words, we considered the similarities to a reference panel as variables. Then, the outcome is predicted from these similarities to a reference set.

### Data

Results on the impact of using individual graphs or not are based upon publicly available real-life data generated by the TCGA Research Network^54^ (https://www.cancer.gov/tcga). We focused on three use cases: prostate cancer severity using the Gleason score, Brain low-grade gliomas (lgg) versus Glioblastoma multiforme (gbm) differentiation, and lung adenocarcinoma (luad) versus lung squamous cell carcinoma (lusc) differentiation. An overview of the data structure is presented in Table 1. For each use case, we analysed two data modalities: RNA-Seq data and histopathology Whole Slide Images (WSI). Previous work motivated the choice of images and genomic data, showing their high potential to disentangle the complex mechanisms of cancer^6,21,40,43,57^.

Prostate cancer is the most common cancer in men^14^, and prostate cancer stages are commonly described according to the Gleason Score, which helps evaluate the prognosis^3,12,55^. This score is derived from the appearance of cancerous cells that can correspond to 5 patterns (normal to tumour cells). Grade 1 cells do not differ from normal prostate tissue; grade 5 corresponds to tumour cells. Thus, cancers with a higher Gleason score are more severe. Physicians determine the Gleason score by looking at biopsy samples and assigning one grade to the predominant pattern (primary Gleason score). Usually, a second Gleason grade is given to the second most predominant pattern, and the two grades are added to set the secondary Gleason score. This project focuses on the primary Gleason score and specifically on patterns 3 and 4. Indeed, the differentiation between Gleason score 3 and Gleason score 4 may play a pivotal role in understanding the inherent prognosis associated with these distinct biological behaviour patterns, and aid in guiding the translation of findings from molecular and histological levels into more precise treatment selection^4,33^ (details in supplementary). In this work, we examine if our workflow can highlight the differences between these two patterns. The database contains 297 individuals in the training set (130 patterns 3, 167 patterns 4) and 71 in the testing set (34 patterns 3, 37 patterns 4).

Brain low-grade gliomas (lgg) are cancerous brain tumours. They arise from the support cells in the brain. Glioblastoma multiforme (gbm) is an aggressive cancer in the brain or spinal cord. Studies have already identified variations between these two tumours, such as gender-specific molecular differences^28^. Here, we study if INs and combining RNA-Seq and histopathology data can help identify these two brain tumours. The training set contains 344 individuals (282 lgg, 62 gbm), and the testing set 156 individuals (122 lgg, 34 gbm).

Lung adenocarcinoma (luad) and lung squamous cell carcinoma (lusc) are among the most common lung cancer subtypes and are both considered non-small cell lung cancer (NSCLC). They have different biological signatures, but these variations in their biological mechanisms remain to be disentangled even though recent studies have made progress^7^. The training data contains 603 patients (232 luad, 371 lusc), and the testing data has 140 patients (50 luad, and 90 lusc).

Feature extraction is performed on the histopathology Whole Slide Images (WSI). We considered the full TCGA dataset with 30 cancer types (See supplementary Table 1), including the types to classify in the 3 use cases described above (i.e., brain, lung, and prostate cancers), but excluding the individuals in the testing sets. A pretrained neural network model is applied to differentiate between the cancer types. In particular, we use Resnet18^19^ and attention MIL^24^, trained for 100 epochs on all TCGA slides, sampling 128 random tiles per slide every epoch. The 512 features contained in layer N-1 are selected as new variables. Each feature is a vector of length the number of individuals and contains discriminative information for cancer type. We assumed that the difference in cancer types would provide relevant information for differentiating the groups in our 3 use cases.

**Table 1:** Data structure.

	Prostate cancer	Brain cancer	Lung cancer
Classes	Gleason score 3/4	lgg/gbm	luad/lusc
Patients in training set	130/167	282/62	232/371
Patients in testing set	34/37	122/34	50/90

### Single data source

#### Level of individual nodes

We created baselines where only the node weights of the individual networks are used, i.e., only the raw feature values. We will refer to them as *node level* approaches. This approach can be considered not relying on individual network structures as the interactions between features are not used in the model. Three ways of obtaining a Person-to-Person network $PPN_n$ with these individual graphs were tested. $PPN_n(x,y)$ indicates how similar individuals x and y are.

The first option was to use the euclidean distance between each pair of individuals’ features and to compute the affinity matrix that represents the neighbourhood graph of the individuals using the function *affinityMatrix* in package *SNFtools*^52^. This function takes three arguments: a distance matrix (obtained with the euclidean distance), a parameter *K*, and $\sigma$. *K* is the number of neighbours, where affinities outside of the neighbourhood are set to zero, and affinities inside are normalised. $\sigma$ is a hyperparameter for the scaled exponential similarity kernel used to conduct the actual affinity calculation. These parameters were chosen empirically ($K=20$, $\sigma =0.05$).

A variation was to apply the Gaussian kernel via the function *gausskernel* from the package *KLRS*^13^. Given two vectors $v_x$ and $v_y$ of descriptive variables for individuals *x* and *y*, the Gaussian kernel is defined as $k(x,y)=exp(\frac{-|| v_x - v_y ||^2}{\sigma ^2})$ where $||v_x - v_y||$ is the Euclidean distance and $\sigma ^2$ (here, $\sigma =1000$) is the bandwidth of the kernel.

The third option was to compute the Spearman correlation between each pair of patients using the function *rcorr* from package *Hmisc*^18^.

#### Level of individual edges

A second type of Person-to-Person network $PPN_e$ was computed to measure the impact of considering the interactions between variables. Specifically, we built a network for each individual where nodes are variables and edges represent the link between these variables.

Because individual networks can be very large when the number of variables per data source increases, we performed feature selection at the node and edge levels. This strategy allowed us to focus on relevant signals and to decrease the computing time. Since a change in the node and edge selection can produce different final results, our pipeline integrates the optimization of the feature selection process, ensuring an efficient network sparsification. The feature selection process unfolded as follows. First, we selected data features (i.e., nodes) with the highest standard deviation. The aim was to evaluate the inclusion of the first 100, 200, ..., to 1400 genes. Then we performed the edge-level feature selection. We created condition-specific networks for each subgroup to predict. We calculated the difference between these condition-specific network adjacency matrices and selected edges with the largest absolute differences. Namely, we evaluated the edge weights percentile thresholds 0.25, 0.5, and 0.75. To select the optimal thresholds, we performed a stratified 5-fold cross-validation within the training set and chose the parameters giving rise to the higher macro F1 score on average.

We derived individual edge weights based on two approaches. In the first one, which we called *Node Product*, we applied a minimum-maximum normalisation algorithm across all variable values to scale them between 0 and 1: $i'=\frac{i-min(i)}{max(i)-min(i)}$, with *i* and $i'$ two variables. Then, for an individual *x*, the weight of the edge between nodes *i* and *j* is $e^x_{ij}=i'^x \times j'^x$. Since this method is computationally efficient, this is the one used to build INs for feature selection. The second approach to create edge weights was the *LIONESS* algorithm^30^. The general idea is to study the difference between a network constructed from all individuals and a network derived from all but one individual. If a difference appears, it must be due to the individual being left out. The *LIONESS* equation is the following: $e^x_{ij}=N(e^\alpha _{ij}-e^{\alpha -x}_{ij})+e^{\alpha -x}_{ij}$, where $e^{\alpha }_{ij}$ is the weight of an edge between nodes *i* and *j* in a network modeled on all *N* samples and $e^{\alpha -x}_{ij}$ is the weight of that edge in a network modeled on all samples except the sample of interest *x*. For each individual, we derived edge weights using the lionessR function^31^. Notably, these edge weights are specific to the reference panel. We reduced the INs obtained to the previously identified selection of edges. Finally, we applied the minimum-maximum scaling algorithm on INs so that the edge weights range between 0 and 1. In particular, we considered the minimum and maximum weights across all INs in the scaling so that the ordering of the weights remained the same between individuals. We used the similarities to individuals in the training sets as new variables for prediction.

Using as the predictor variable, how similar individuals are from a reference panel requires defining a measure of distance between individuals. Many methods have been developed to compare graphs. The specificities of our context limited the choice of distance. Indeed, the measure should be computed in a reasonable amount of time, even on large graphs, and should handle undirected and weighted networks. Since the same variables (e.g., same genes) are used for all the individuals, there is a node correspondence between the different INs (multiplex). Only the edge weights differed from one person to another. That implies that without additional filters, all the graph distances based on structural differences will not allow us to identify if some graphs are more similar than others. For example, the euclidean, Jaccard, edge difference, or DeltaCon distances suited our context. We used the edge difference distance in this project because of its good computational properties. This distance takes two adjacency matrices and computes the Frobenius norm of their differences. We applied this distance to each pair of individual networks to obtain a matrix of similarity between individuals.

#### Combination of individual nodes and individual edges

There is no reason to assume that both individual node and edge information could not be complementary. Thus, we also investigated the combination of Person-to-Person networks built from individual nodes and individual edges. We built the Person-to-Person networks independently for the two approaches, and we averaged their corresponding adjacency matrices to merge them.

### Data integration

One option to integrate multiple database information was concatenating the original data (early fusion) and applying the pipeline as in the single data procedure. When using individual edges, or edges and nodes, we included the additional step: for each data source, the top variables are selected as described in Sect. “Level of individual edges”. Multiple edge correlation thresholds (0.25, 0.5, and 0.75) are tested to reduce the INs further. Here, nodes can be variables from any of the original databases, and edges can therefore represent the association between any variables.

Fusion can also be performed at the PPN level (intermediate fusion). PPNs are obtained for each data source separately and merged to benefit from their potential complementary. Simple methods can be applied for this task, such as computing the average of the different PPNs. More advanced approaches include the Similarity Network Fusion (SNF)^53^. SNF has proven efficient in combining multiple data such as mRNA expression, DNA methylation and microRNA expression data for cancer data. In this project, we tested both the average and the Similarity Network Fusion algorithms. Then, an SVM model was applied as described in Sect. “Prediction and performance assessment”.

The last alternative considered was late fusion, where data are merged after an independent investigation of each data source (a and b). With continuous outcomes, it can be computed by summation or averaging. Since we are performing classification, we used the majority vote approach. In most of our application settings, we considered two data modalities only, so a majority vote would not provide additional knowledge. Hence, we applied late fusion only on results obtained from Person-to-Person Networks derived from the combination of individual nodes and edges, where we consider prediction from four outcomes: $PPN_{n,a}$, $PPN_{n,b}$, $PPN_{e,a}$, and $PPN_{e,b}$. When two labels were predicted equally for an individual, the final label was randomly assigned to one of them.

### Prediction and performance assessment

The Person-to-Person Networks were normalised with the following transformation: $PPN(x,y)=\frac{PPN(x,y)}{\sqrt{PPN(x,x)PPN(y,y)}}$. They were used to train Support Vector Machines (SVM) using the *kernlab* package^25^. Instead of working with the original sample representation in the original dimensional space, SVM classification methods operate directly on similarity matrices. We tested multiple options of C ($10^k$ for $k=1..5$), the cost of constraints violation^25^. We selected C giving rise to the best performance. We obtained the performance by comparison with the ground-truth labels. As the groups are unbalanced, we used the macro $F1\,score$ to assess the performance, with Macro $F1\,score=\frac{1}{L}\sum _{l=1}^{L}F1\,score_i$, where *l* is the label index and *L* the number of labels.

### LIMMA and gene set enrichment analysis on graphs

We associated the prediction methodology described above with complementary approaches, such as LIMMA^30^ and pathway analyses^42^ to showcase the advantages of using individual graphs.

Originally, LIMMA is an analysis of gene expression data that uses linear models to simultaneously assess differential expressions between many targets. We conducted LIMMA analysis on RNA-Seq individual networks, because genes are interpretable units of analysis. Our input consisted of the individual networks constructed using *LIONESS* and reduced to selected nodes and edges as previously defined in Sect. “Level of individual edges”. Specifically, the individual networks are presented in a matrix *M*1 where columns correspond to individuals and rows to gene pairs. Additionally, the matrix *CT* summarizes the cancer type to which each patient belongs. Our analysis proceeded as follows.

First, we applied the *lmFit* function from the R package limma^42^ to (*M*1, *CT*). This function fits a linear model for each gene-pair and returns a MArrayLM object, denoted as *fit*, containing the results of these fits. Subsequently, we applied the *contrasts.fit* function on *fit* to compute estimated coefficients and standard errors for each linear model. Next, the *eBayes* function was applied to the output of the *contrasts.fit* function to compute moderated *t*-statistics, moderated *F*-statistics, and log-odds of differential expression using empirical Bayes moderation to adjust standard errors towards a common value. Finally, we used the *toptable* function to extract a matrix containing the top-ranked gene-pairs based on the linear model fit. We focused on identifying the top 50 most differentially co-expressed gene pairs and colored the edges in the network based on their values within the classes to predict. This approach highlighted edges with significantly distinct weights between different groups, thus enhancing our comprehension of the underlying data. In parallel, we also conducted a LIMMA analysis to assess significant differences in gene expression levels between groups. Hence, nodes in the network were colored based on the t-statistics resulting from the LIMMA analysis.

We investigated networks obtained with LIMMA with a gene set enrichment analysis^17^. We focused on the largest connected components. Since all the genes in that module are connected, they can indicate a broader biological mechanism responsible for the group difference. For the pathway analysis, we performed the LIMMA analysis on the features selected as described in Sect. “Level of individual edges”, and we used the *fgsea* package^45^ to perform gene set enrichment analysis with a minimum gene size of 10 and 5000 permutations. Two inputs were required: a ranked gene list and a list of gene sets to test for enrichment. For the former, we used the gene *t*-values of the genes in the largest component from the LIMMA analysis. It represents the gene statistical difference between the two groups compared. For the latter, we downloaded all ontology and curated Molecular Signature Database (MSigDB version 7) gene sets^34,50^. We applied FDR cut-off of 0.05 for significant assessment.

### Model comparison

We compared our graph-based approach to multiple classification methods applied to the raw features. Namely, we used a penalized logistic regression, a classification tree, a random forest, AdaBoost, a naive Bayes method, and a neural network approach. The algorithms were applied on each data type separately (RNA-Seq and histopathology features) and on the combined dataset (RNA-Seq and histopathology features concatenated). For each algorithm, we computed the associated macro $F1\,score$ to show how our model and its variants compare to standard and state-of-the-art classification methods. Details about the algorithms and the options applied can be found in the supplementary material. Note that these five models are only compared to the graph approaches based on IN’s edges, and IN’s nodes-and-edges, as the approach based on IN’s nodes is not using any graph structure in the process.

## Results

### Single data-source: Exploiting edges in the individual graphs enhances classification

We compared the prediction performance obtained using different levels of information in the individual graphs. To create INs, feature selection is performed as described in Sect. “Level of individual edges”. It gave rise to a selection of 700 genes (edge weight percentile threshold $t_{edge}=0.25$) and 300 histopathology features ($t_{edge}=0.25$) for the prostate use case. In the brain cancer dataset, 600 genes ($t_{edge}=0.5$) and 300 image features ($t_{edge}=0.75$) were retained. For the classification of the two lung cancers, 600 genes ($t_{edge}=0.5$) and 500 histopathology features ($t_{edge}=0.75$) were considered.

**Figure 2:**
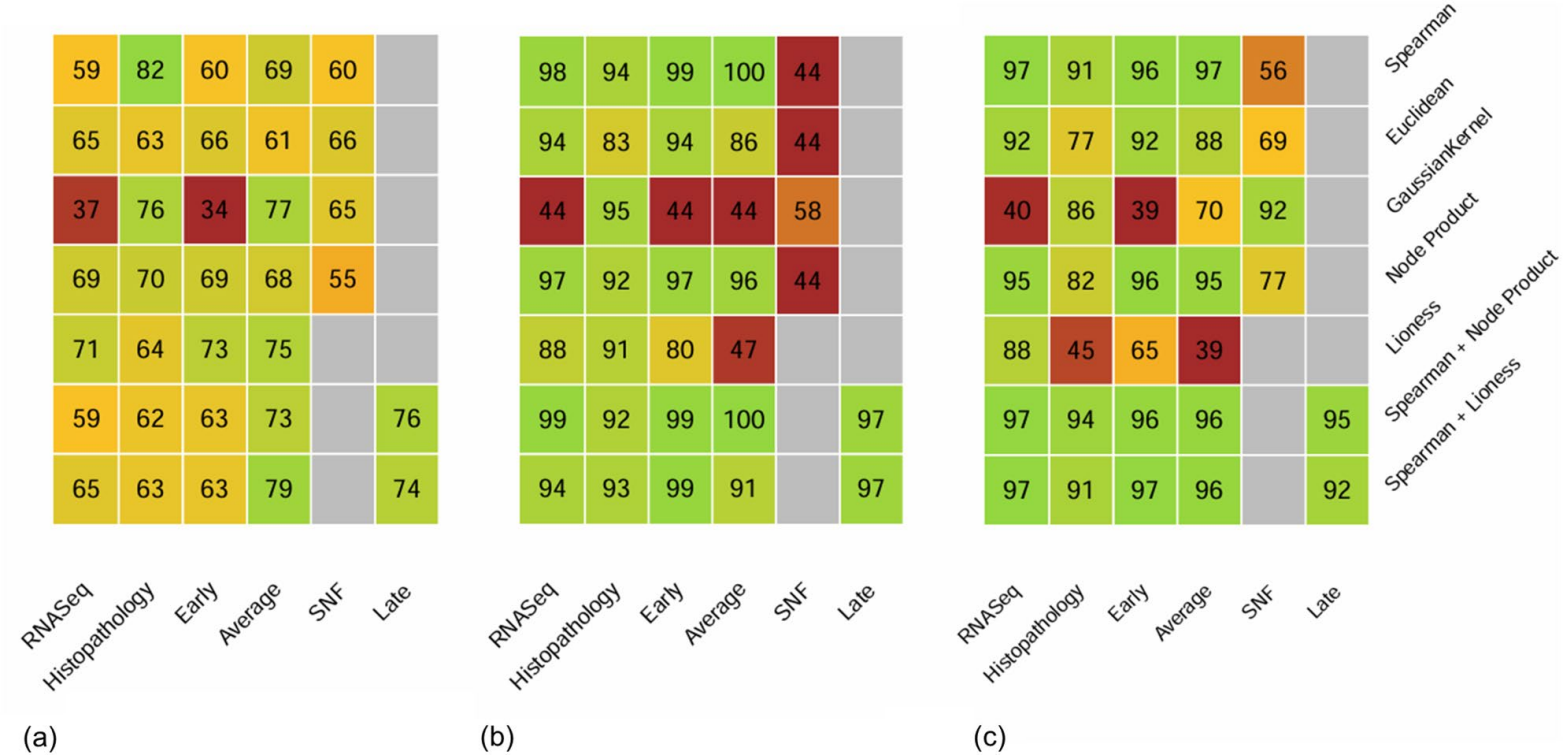
Overview of the macro F1 scores (%) for the different multi-modality approaches for the prostate cancer severity (**a**), types of brain cancers (**b**) and types of lung cancer **(c)**, described in 2.1. The greener, the better the prediction, and the redder, the worse the prediction. Untested approaches are grayed out. The three first rows refer to approaches based on the nodes of the individual networks. Rows 4 and 5 use the edge weights of the individual networks. Rows 6 and 7 combine individual nodes and edges. The two first columns focus on a single data modality. Columns 3 to 6 refer to data integration.

The first two columns of each heatmap (Fig. 2) show the effects of using the nodes (rows 1 to 3), edges (rows 4 and 5), or nodes and edges (rows 6 and 7) of the individual network on each data modality. Additional visualisation is presented in the supplementary Figure 6. The Spearman correlation performed best in two-thirds of the scenarios among the three methodologies to build similarities at the *node level*. It motivated the choice of the Spearman correlation for the combination of *node level* and *edge level* information.

We observed that using more than node information increased the macro F1 score for the prostate (max F1=0.71) and brain (max F1=0,99) use cases with RNA-Seq data, and for the lung use case (max F1=0.94) with histopathology data. Using individual edges or individual nodes led to an equal performance in the context of lung cancer (max F1=0.94) from RNA-Seq data. Pipelines based on *node level* information achieved higher prediction with histopathology data for the prostate (max F1=0.82) and brain classifications (max F1=0.94).

Among the two approaches to build similarity matrices via individual edges, the *Node Product* performed better than the *LIONESS* algorithm in all situations except the prediction of prostate cancer severity using RNA-Seq data. However, when combining individual nodes and edges, the *LIONESS* method yielded higher results in half of the situations. In general, on single data, classification based on individual edge weights, with or without combination with individual node weights, was better or equal to predictions from individual nodes only (i.e. no individual graph structure) in two-thirds of the scenarios.

**Figure 3:**
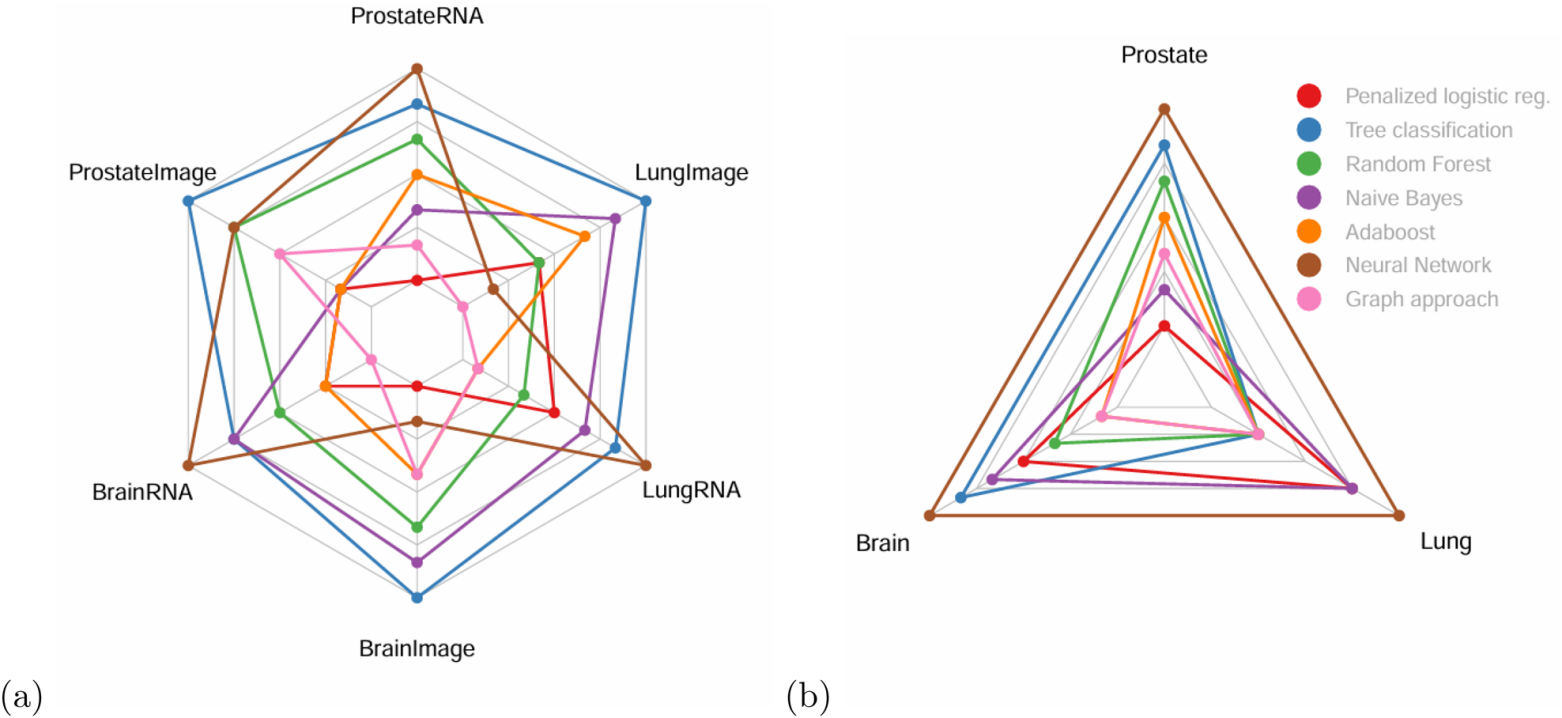
Comparison of the graph-based models to multiple classification algorithms applied to the raw features. Models are ranked according to their prediction performance. The lower the area in the colored lines, the better. (**a**) shows the average rank of each model across datasets (prostate, brain, lung) and data types (RNA seq or histopathology). (**b**) shows the average rank of each model across datasets for the combined (i.e. concatenated) data types. For each analysis, the best graph approach is presented. Since the method based on IN’s nodes is not using any graph structure information, only the approaches based on IN’s edges, and IN’s nodes-and-edges are considered as *graph-approaches*.

Moreover, we compared graph-based models to multiple classification algorithms applied to the raw features, separately for each data type: a penalized logistic regression, a classification tree, a random forest, AdaBoost, a naive Bayes method, and a neural network approach. The data were pre-processed following the same approach as in the graph method, specifically selecting the same variables. Results are detailed in Table 2. Then, the models were ranked based on their macro F1 scores, with the best model ranked 1 and the worst model ranked 7. The ranks are presented in Fig. 3a, where a lower the area in the colored lines indicates better performance. Among the six analyses conducted, the graph-based approaches outperformed the other models in three of them. Overall, the graph-based approaches achieved the best results, with an average rank of 2.2. Following closely, the penalized logistic regression achieved the second-best performance, with an average rank of 2.3. These results demonstrate the substantial potential of individual graphs for disease subtyping.

**Table 2:** Macro F1 scores (%) for the different inputs (RNA-Seq data only, histopathology images only or fusion of the two modalities) and algorithms evaluated.

		**Prostate**			**Brain**			**Lung**	
	RNA-Seq	Histo.	Fusion	RNA-Seq	Histo.	Fusion	RNA-Seq	Histo.	Fusion
Penalized log. reg.	76	74	83	97	95	98	90	89	95
Tree classification	63	57	57	92	79	88	96	77	96
Random forest	64	69	72	95	91	99	95	89	96
Naive Bayes	70	74	82	92	80	96	93	83	95
Adaboost	69	74	78	97	93	100	96	87	96
Neural network	53	69	47	75	94	71	77	90	75
*Graph approach*	71	70	79	99	93	100	97	94	97

### Multi-modality integration: Intermediate fusion demonstrates superior performance

This analysis’s first goal was to study whether it is possible to predict disease subtypes and severity from the patient data using graphs. The results showed that in brain cancer, the workflow achieved perfect predictions. We also obtained high performances in the lung cancer use case ($macro \, F1 \, score=0.97$). The severity of the prostate cancer was more cumbersome to detect, with a maximum macro F1 score of 0.82.

The second goal of this study was to examine which modality yields the best prediction. The answer differs depending on the use case. The histopathology images were the most informative data in the prostate scenario, but the RNA-Seq data achieved better results for the lung and brain scenarios.

The third aim was to leverage the consequences of using INs and PPNs to combine database information at various steps. The impact of multi-modality integration using the edge weights of the individual graphs is shown in rows 4 and 5 of each heatmap (Fig. 2). An alternative visualisation is presented in the supplementary Figure 7. With the Person-to-Person Networks derived from individual edge weights only (i.e., no node weights), the fusion of the two data sources provided better results for the prostate (max $F1=0.75$) and the lung (max $F1=0.96$) cancers. There was no difference between one modality or the fusion of two modalities for brain cancer (max F1=0.97). Hence, with Person-to-Person networks derived from individual edge weights only, there was a benefit of combining multi-modalities, although there was no clear outperformer between the *LIONESS* and the *Node Product* methodologies.

When considering Person-to-Person networks computed from the combination of individual edge weights and individual node weights (Fig. 2, rows 6 and 7 of each heatmap), the fusion of the RNA-Seq and histopathology data produced improved predictions for the prostate (max F1=0.79) and the brain cancers (max $F1=1$). There was no observed difference for lung cancer since multiple pipelines produced the best macro F1 score of 0.97. Thus, with the combination of individual node and edge weights, we also observed that performance was improved in two-thirds of the situations with the fusion of the two data sources.

In all three cancer use cases, the average intermediate fusion technique consistently achieved the highest predictions. For lung cancer, early integration provided comparable results. The primary drawback of late fusion lies in the inability to capture interaction effects between RNA-Seq variables and histopathology features, as these two modalities are never jointly considered within the same model. Early data integration, if not handled appropriately, can exacerbate the issue of high dimensionality in the input space and potentially introduce noise, which can detrimentally impact model performance. While dimensionality reduction methods ideally aim to select the most informative features, their effectiveness may vary in practice. Our results highlight that, in many scenarios, the intermediate fusion approach based on the average of the PPNs emerges as a preferred approach due to its ability to consistently deliver superior results.

Finally, we compared results across all analyses: use of nodes and/or edges in the individual graphs, and use on one or two data modalities (entire heatmaps - Fig. 2). For prostate cancer, the two best results were obtained from the Spearman correlation on histopathology data and the average intermediate fusion of the two data types with the combination of *node level* and *edge level* information. For the brain cancers, the best results were achieved via the average intermediate fusion of the two data modalities with Spearman correlation and via the intermediate fusion with the combination of *node level* and *edge level* information. Note that the brain classification was already perfect ($macro \, F1 \, score=1$) with *node level* information only, and there was, therefore, no possible improvement with individual edges. An ideal use case would require complementary data, each one bringing partial information. For lung cancers, the maximum macro F1 score was obtained from six different settings involving the Spearman correlation and the combination of Spearman correlation and approaches based on individual edges. Hence, no approach outperformed the others in all contexts, and no general rule could be derived.

In addition, we conducted further analyses to assess the added value of our graph-based models (Table 2). Specifically, we compared these models to several classification algorithms applied to the raw features on the combined data types, where the features from the different data types were concatenated. Data were pre-processed as in the graph approach. The outcomes of these analyses are displayed in Fig. 3b. The graph-based approaches performed best in the brain and lung analyses. Hence, when considering the overall performance, the graph-based approaches exhibited the best results, with an average rank of 2.3. Then, AdaBoost, Random Forest and the penalized logistic regression algorithms achieved the next-best performance, with an average rank of 2.7, 3.5 and 3.5 respectively. These findings further underscore that the graph-based models provide valuable insights and demonstrate their effectiveness in handling combined data types.

### Contribution of data modalities and their impact on misclassification

We emphasize the contribution of each data modality to the classification performance in the context of late fusion in Table 3. We observe a notable agreement between modalities, often resulting in high scores even during single-data analysis. In most cases, the relative contribution of each modality to late integration results aligns closely with their individual performance. In other words, when the single-modality prediction performs well (as seen in columns 1 and 2 of Fig. 2), its contribution to the two-modality prediction is also substantial. We have found that histopathology images contribute more significantly to the predictions than RNA-Seq data in prostate classification. For brain classification, RNA-Seq data and histopathology images make nearly equal contributions. For lung cancer, RNA-Seq data contributes more than histopathology images, indicating variability in the modality impact across different cancer types.

Determining the precise contribution of each modality to the classification performance in early and intermediate fusion is a complex task due to the multi-step nature of these approaches. In both early and intermediate fusion, the input for the Support Vector Machine consists of a Person-to-Person Network already containing combined information from the two data modalities. Therefore, our analysis primarily allows us to discern the relative improvement gained from using one modality versus both. For instance, in the case of employing both nodes and edges of the Individual Specific Networks using the *Node Product* approach (as depicted in Fig. 2, row 6), we observe the following performance metrics: an F1 score of 0.59 with RNA-Seq data only, 0.62 with histopathology data only, and a notable increase to 0.73 with intermediate fusion. This comparison highlights the relative enhancement achieved through the integration of both modalities, showcasing the benefit of utilizing combined information.

Misclassified patients can arise due to several factors such as a class imbalance in the training set. The potential bias towards specific cancer subtypes was evaluated by comparing the class-wise F1 scores (Supplementary Table 2). Our analysis with the graph-based approach revealed an average difference of 3.3% between the F1 scores of the different cancer subtypes (e.g. lgg/gbm), with two analyses showing equal F1 scores for both classes. The largest discrepancy (11%) was observed in prostate cancer classification based on RNA-Seq data. These results indicate that there is no substantial disparity between the F1 scores achieved in the two groups, and suggests that the approach adeptly handles data imbalance. Furthermore, we observed no discernible patterns associated with genes or histopathology features among the misclassified samples. In some cases, the characteristics of different cancer subtypes might overlap, making it challenging to differentiate between them. In our study, this phenomenon becomes particularly relevant in the context of prostate cancer, where Gleason scores are derived manually by doctors. This can result in ambiguous cases that are prone to misclassification.

The dataset’s size may also play a role in misclassifications. We are training our model on relatively small datasets (maximum 603 patients), so the model may struggle to capture the full spectrum of variation, leading to errors. Data noise in RNA-Seq or histopathology data can also misguide the algorithm, potentially arising from data collection, preprocessing, or annotation errors. Incomplete information in the dataset, missing important features influencing cancer subtype, can contribute to misclassifications as well. Therefore, including additional data modalities could reduce the number of misclassified patients. Finally, the algorithm’s complexity might not be perfectly-suited to the dataset. Overly complex models can overfit the data and lead to misclassifications. For instance, in the case of prostate cancer, while node information from histopathology data provides valuable insights for disease subtyping (macro F1 score of 0.82), the edge information lacks meaningful contributions and even hinders predictions (maximum macro F1 score of 0.70).

**Table 3:** Contribution (%) of the RNA-Seq data and the histopathology images to the classification performance in the context of late fusion. The frequency with which the modality-specific prediction aligns with the final prediction is indicated. Indeed, if the class-wise prediction is not in the majority, it does not impact the final prediction outcome. Instances where there are equal votes for disease subtypes 1 and 2 were omitted from this calculation since the class assignment is random in such cases. N (resp. E) indicates the score derived from the PPN based on node (resp. edge) information only.

	**Prostate**		**Brain**		**Lung**	
	RNA-Seq	Histo.	RNA-Seq	Histo.	RNA-Seq	Histo.
LIONESS (N/E)	84 (76/93)	90 (96/84)	99 (98/99)	98 (99/97)	99 (100/99)	93 (98/88)
Node Product (N/E)	79 (69/87)	92 (92/92)	97 (99/95)	96 (97/96)	99 (100/98)	85 (90/74)

### Advantages and limitations of workflow variations

The predictive capabilities of data modalities in cancer classification is influenced by their inherent informativeness, and their capacity to complement each other. In the case of prostate cancer, the high performance of histopathology images was expected since primary Gleason scores are typically assessed through biopsy samples. This data modality inherently captures valuable information essential for subtype classification. Still, when the Person-to-Person networks generated from both the individual edge weights and individual node weights (Fig. 2a, rows 6 and 7) were considered, the fusion of RNA-Seq and histopathology data yielded improved predictions for prostate cancer (F1=0.79) compared to single-modality predictions (0.65 and 0.63). This suggests that the two modality types contain complementary information that, when combined, can enhance predictive power. For brain cancers, the two modalities already exhibited strong predictive capabilities individually, indicating that both the gene expression and the histopathology data can help differentiate the brain low-grade gliomas and glioblastoma multiforme. However, the highest performance was achieved by integrating both modalities. Table 3 underscores that both data types contribute to late fusion prediction, further supporting the value of integration in this context. In the context of lung cancer, RNA-Seq data proved slightly more predictive than histopathology, although both data types made substantial contributions to the performance. The macro F1 score was not improved by combining the two data types. Therefore, our results suggest that either histopathological data did not provide additional information compared to gene expressions, or the graphical model did not effectively exploit this second type of data to improve prediction accuracy.

We evaluated the benefits of using nodes, edges, or nodes and edges in the workflow. In the context of brain and lung cancers, the highest macro F1 scores were achieved when considering nodes, either alone or in combination with edges. This suggests that the superior performance of the latter configuration primarily arises from the contribution of individual nodes. However, we observed more heterogeneous performance outcomes when exclusively focusing on nodes or edges, compared to the results when incorporating both nodes and edges. For instance, with lung cancers, the utilization of node-based information (Spearman) yielded an average macro F1 score of 87.5 across all possible data type combinations, including RNA-Seq only, histopathology only, early, intermediate, or late integration. When both nodes and edges were incorporated, the average macro F1 score increased to 95.1. We observed similar results with brain cancers. These findings underscore the significance of including both nodes and edges information, as it reduces the risk of achieving very low performance results and enhances the overall predictive stability.

We compared the two types of intermediate fusion: SNF versus the average approach. The Similarity Network Fusion approach offers the advantage of capturing complex relationships between data sources, which can be especially valuable when dealing with multiple modalities. It can model dependencies that might be overlooked by simple averaging. However, in our specific analysis, we only considered two data modalities, which might explain why the average method outperformed SNF in most cases. The average approach, while simpler, can still deliver strong results when dealing with a limited number of modalities. It might be less suitable when complex interactions exist between numerous data sources. One drawback of averaging is that it assumes equal importance for all data sources, which may not always hold true. This could account for the reduced performance of the average approach in the context of prostate cancer, given that the predominant predictive strength originates from histopathology images. On the other hand, SNF can be computationally demanding, particularly with large datasets or when dealing with numerous data sources. It also requires defining parameter values, such as determining the number of nearest neighbors. This parameter selection process can introduce complexity and affect the results. Hence, we recommend considering SNF in the presence of intricate relationships between multiple data sources. However, for scenarios with a limited number of modalities, average fusion remains a simpler, computationally efficient option that can still yield higher performance.

### Interpretability: graphs identify biological processes underlying the differentiation of cancer subtypes

We applied a LIMMA analysis and a gene set enrichment analysis to the RNA-Seq data to illustrate the potential of individual networks to understand biological mechanisms.

**Figure 4:**
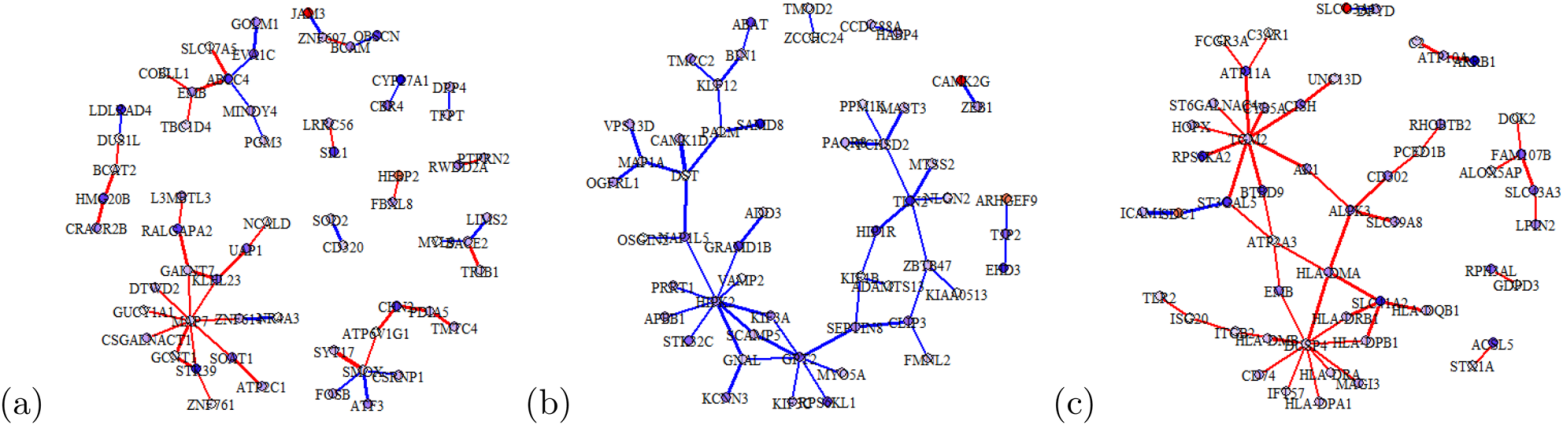
LIMMA analysis on the top 50 most differentially co-expressed edges between groups. Genes with absolute *t*-statistic $<1.5$ are shown in white. In the prostate use case (**a**), edges/nodes are red if they have higher coefficients in the Gleason pattern 3 group (blue for pattern 4). In the Brain use case (**b**), edges/nodes are red if they have higher coefficients in Brain lower grade glioma (blue in glioblastoma multiforme). In the Lung use case (**c**), edges/nodes are red if they have higher coefficients in Lung adenocarcinoma (blue in lung squamous cell carcinoma). Thicker edges represent higher log-fold changes.

To detect which edge weights were significantly different between the classes (e.g., Gleason score 3 versus 4), the top 50 most differentially co-expressed edges were selected and coloured (See Fig. 4a-b-c). Nodes with significant gene expression differences between groups were also identified based on the *t*-statistic from the LIMMA analysis (See Sect. “LIMMA and gene set enrichment analysis on graphs”). This visualisation gave a general overview of the organization of the most relevant gene pairs differentiating between groups while highlighting specific nodes and interesting modules. We can, for instance, investigate the most connected genes (more than 5 connected neighbours). The Gleason score classification pointed MAP7, which is prognostic for survival in patients with stage II colon cancer^2^. In brain cancer prediction, GTP2 and HIPK2 were identified. GTP2 is linked to neurological disease, encephalopathy, and microcephaly^20,27,41^, and HIPK2 is associated with tumor progression, and malignant neoplasm^15,56^. In lung cancer differentiation, we detected TGM2 and DUSP4. A loss of DUSP4 is observed in EGFR-mutant tumours^8^. Hence, the graph approach helped target gene and gene pairs differentiating between the two investigated groups.

We performed the gene set enrichment analysis described in Sect. “LIMMA and gene set enrichment analysis on graphs” to investigate the biological mechanisms associated with the difference between subtypes. It was based on the LIMMA analyses that include all the features identified in Sect. “Single data-source: Exploiting edges in the individual graphs enhances classification”. Namely, 700 genes (edge weight percentile threshold $t_{edge}=0.25$) are investigated for prostate cancer, 600 genes ($t_{edge}=0.5$) for brain cancer, and 600 genes ($t_{edge}=0.5$) for the lung cancer. 36 gene sets are enriched in the prostate cancer use case (See supplementary Figure 8) and 10 gene sets in the lung cancer use case. No enriched pathway was detected for the two types of brain cancers. The most significant gene sets for the prostate analysis was the *Chandran metastasis*. In prostate cancer, metastasis represents the most adverse outcome, and it is assumed that genes associated with this pathway have a role in the biology of metastatic disease^5^. We also identified the *Liu prostate cancer* set^35^ that is linked to a study showing that sex-determining region Y Box 4 is a transforming Oncogene in human prostate cancer cells. In the lung cancer scenario, the most significantly enriched gene set was *Shedden lung cancer good survival a4*^46^, coming from the investigation of gene expression-based survival prediction in lung adenocarcinoma. Thus, these results highlighted the relevance of graphs in identifying biological processes involved in differentiating cancer subtypes.

## Discussion

Despite the increasing volume of human data, methods for data-modality integration are understudied. Commonly, late integration is performed manually and relies on prior knowledge of the disease studied. Moreover, biological mechanisms are often organized as complex systems. Allocating a network to each individual could model such interactions while accounting for individual specificities. Starting from these observations, we investigated the added value of individual graphs for cancer subtyping. We integrated data in the space of individuals rather than measurements (e.g., gene expressions) using networks of similarities between patients. First, we evaluated the benefits of transforming the input data into individual graphs on single data. We showed that considering the features as a connected system could improve the prediction performance. Second, we demonstrated that combining individual networks and multi-modality integration can yield better performance. Finally, as we illustrated with cancer data, one strength of graph-based approaches is the ability to visualise and provide insights into the causal factors accounting for the differences between the disease subtypes. Although we focus here on RNA-Seq and histopathology data, our framework applies to any multiplex data. In clinical studies, it offers opportunities to integrate various measurements such as demographic, microbiome, and metabolomics data.

### Suggested guidelines for the proposed workflow

As any machine learning algorithm, the performance of the graph-based models on other datasets that might lack uniform processing can be influenced by several factors, such as sequencing depth, batch variation, and unknown combinations of technical and biological noise. To mitigate the impact of these challenges, our approach incorporates various strategies.

We employ a dataset-specific feature selection step at both the node and edge levels of the individual networks within our workflow. This process optimizes feature selection, promoting an efficient individual network sparsification. By focusing on the most informative features, which are less susceptible to noise, we aim to enhance model performance. Also, our pipeline incorporates cross-validation techniques, particularly when applying the Support Vector Machine on the Person-to-Person network. Cross-validation assesses the model’s performance across multiple data subsets, thereby helping identify potential overfitting to a single dataset.

In SVM, the regularization parameter C is the cost of constraints violation^25^. It essentially determines the balance between two objectives: the minimization of training error and the minimization of model complexity^10^. Specifically, opting for a higher value of C results in a more flexible model, characterized by a narrower margin, and fewer misclassification. Conversely, a lower C value allows for a wider margin, potentially leading to more misclassifications within the training dataset. We selected C giving rise to the best performance. Feature selection and hyperparameter tuning are performed on the training set, presenting a potential risk of overfitting in the intermediate results. Also, selecting an algorithm based solely on its performance evaluation on a test set, after training on a training set, could introduce potential overfitting concerns. We acknowledge that opting for a separate validation set, instead of the training set, during hyperparameter tuning would enhance the model’s capacity to generalize to new data. Nonetheless, we assess the model’s performance using a distinct test set (Sect. “Data”). Hence, we emphasize that the final results are not overly optimistic, given that the evaluation takes place on an entirely independent dataset.

Based on our findings, it appears that no single variation of the workflow consistently outperforms the others across all scenarios. Therefore, we recommend that users, explore multiple variations of the pipeline to determine which one aligns best with their specific dataset. This way, they can make a more informed decision based on their dataset’s unique characteristics. Also, combining predictions from these variants, each trained on different data subsets or with distinct algorithms (e.g. different metrics or data fusion), can enhance overall model performance and limit the impact of unknown combinations of technical and biological noise. Alternatively, users may consider adopting the construction of Person-to-Person networks, leveraging both individual network nodes and edges in conjunction with an intermediate fusion (average) approach. This strategy demonstrated high performance in multiple scenarios.

Furthermore, by creating UMAP^38^ visualizations for different sets of options or parameter configurations, users can compare the effects of these options on data transformation. We employed UMAP to visualize PPNs and color-coded data points according to their prediction labels. Specifically, we applied the *umap* function from the R package *umap* with default settings. This approach enabled us to assess how well the algorithm’s predictions align with the patterns in UMAP representations, shedding light on when the network approach added value. The association between a discernible group pattern in UMAP visualizations and enhanced prediction performance using SVM is noteworthy. In UMAP plots generated for the prostate use case, most visualizations lack a clear separation between groups, which reflects the challenges in distinguishing cancer severity (supplementary figure 3). Conversely, in the brain cancer plot, options that yield the best prediction performance, such as the combination of nodes and edges information of the IN (the two last rows), exhibit a more distinct division between groups (supplementary figure 4). The same trend can be observed in the lung analysis. For example, the utilization of nodes and edges information from the INs, particularly when based on RNAseq only, early integration, and average intermediate integration, produces discernible patterns (supplementary figure 5). These options, in turn, are associated with more favorable prediction outcomes. UMAP, therefore, can serve as a visual guide for users, facilitating their comprehension of how the algorithm transforms raw data into input for SVM. They can experiment with different options and observe the impact of these alterations on UMAP visualizations for their specific dataset and problem. In particular, when UMAP visualizations unveil well-defined clusters or meaningful patterns within the labels, we recommend considering the utilization of the associated options.

Furthermore, we encourage users to implement appropriate preprocessing steps, such as batch effect correction and noise reduction techniques. These steps can substantially enhance the model’s adaptability to diverse datasets and improve overall performance.

### Feature extraction from histopathology data: increased generalizability with multiple cancer types

In this study, one data type in the cancer use cases was histopathology Whole Slide Images. This data was transformed beforehand to convert images to image features with continuous values. To achieve this transformation, we considered a dataset that included but was not restricted to the individuals of our use cases, and we applied a neural network model to predict the cancer type. From this model, we derived features that discriminate between cancer types. Even though it may seem more straightforward to apply a neural network for each use case and train on the label of interest (e.g., Gleason score), we observed that differentiating among cancer types yielded better results (data not shown). One possible explanation is that the image model trained purely on the groups to identify resulted in a not general enough embedding. Indeed, a more general model on cancer types contains more individuals, which may lead to a better embedding that recognizes essential features.

### Considerations on the inference of individual networks with *LIONESS*

Computing how similar a new individual is to each individual in the training set is straightforward with the *Node Product* approach. However, with the *LIONESS* algorithm, the INs of the reference panel change when we consider a new individual. Also, the derivation of the IN for a new individual depends on this panel. Hence, to create INs for new individuals, we used all individuals from the training set and one new individual at a time. If we directly add all the new individuals to the ones from the train set, we could considerably modify the relations between the INs of the training set. Adding only one individual at a time to the training set minimises the impact on the similarities between individuals from the train. To summarise, with the *LIONESS* algorithm, for each individual *x* from the test set, we created a new database containing the reference panel and *x*, and we constructed the INs for all the patients in this temporary database. From these new INs, we computed the similarity between *x* and each individual in the training set. Hence, the complexity of creating individual networks for individuals in the test set with the *LIONESS* algorithm motivates starting investigations using the *Node Product* approach.

In theory, *LIONESS* offers a significant advantage over the *Node Product* method by leveraging interaction information rather than simply combining node values. However, the *LIONESS *method does come with a higher computational demand compared to the *Node Product* approach. As a result, for the feature selection, we opted for the *Node Product* approach. Therefore, the improved results observed with the *Node Product* method could, at least in part, be attributed to this choice. Nevertheless, the *LIONESS* approach can still yield superior results in certain scenarios. For instance, in the Prostate use case, the intermediate fusion (average) outperformed using *LIONESS*. Additionally, in the Lung use case, when considering histopathology data exclusively, the combination of nodes and edges with *LIONESS* proved more effective than the *Node Product* method. Hence, both approaches have their distinct advantages and disadvantages, and their suitability depends on the specific context and requirements of the analysis.

### Enhancing multi-modality integration through graph properties

Future enhancements include a data integration strategy that takes advantage of graph specificities. In this work, we studied the impact of combining individual graphs and data integration, but we did not use the network characteristics in the integration itself. Data were integrated before the computation of individual graphs (early integration) or after the derivation of similarity matrices (intermediate and late integration). An alternative would be to combine the data within the process of creating individual networks. In Fig. 1, it would correspond to an intermediate integration occurring at the level of the second box (“Individual networks”). For instance, one could develop a method to select predictive features in individual networks obtained with a first database (e.g., RNA-Seq) from a second dataset (e.g., histopathology data). This approach could allow focusing on interpretable variables while including knowledge of an additional database. Second, it would sparse the individual networks, enabling more advanced graph distances to be used to compare individuals. The development of such methodologies was beyond the scope of this paper.

## Conclusion

Whereas research on disease subtyping has received significant attention recently, individual treatment decisions remain a cumbersome issue. Taking advantage of the complementarity of multiple data sources could help provide more precise subtypes. Ongoing research on multi-modality integration mainly considers one variable at a time, ignoring their interactions. Fusion based on individual graphs accounting for these interactions can bring additional information. In this study, we showcased the potential of graph theory. In particular, we underlined the advantages and limits of this approach in the context of prostate, brain, and lung cancers subtyping and severity assessment.

We observed that individual graphs could be beneficial even on single data sources, and we highlighted that intermediate integration was often among the best performers. Graph-based methods achieved competitive performance while bringing additional explainability properties. We identified biologically relevant genes, gene interactions, and pathways for different use cases. The presented workflow is flexible and can readily be applied to other data modalities. The results motivate more research on methodological developments of individual networks for precision medicine.

### Supplementary Information

Supplementary Information.

## Data Availability

The code necessary to reproduce this article’s results and analyses is available on GitHub at https://github.com/DianeDuroux/graph_multimodal_integration. Analyses are based upon publicly available real-life data generated by the TCGA Research Network (https://www.cancer.gov/tcga).

## References

[1] Ash, J. T, Darnell, G., Munro, D., & Engelhardt, B. E. Joint analysis of expression levels and histological images identifies genes associated with tissue morphology. *Nature Commun*, **12**(1), 1–12 (2021). 10.1038/s41467-021-21727-x PMC7952575 33707455

[2] Blum, C., Graham, A., Yousefzadeh, M., Shrout, J., Benjamin, K., Krishna, M., Hoda, R., Hoda, R., Cole, D. J, & Garrett-Mayer, E. et al. The expression ratio of map7/b2m is prognostic for survival in patients with stage ii colon cancer. *Int. J. Oncolo.*, **33**(3), 579–584 (2008). PMC3399116 18695889

[3] Catalona, W. J, & Smith, D. S. 5-year tumor recurrence rates after anatomical radical retropubic prostatectomy for prostate cancer. *J Urol*, **152**(5), 1837–1842 (1994). 10.1016/s0022-5347(17)32397-2 7523731

[4] Chan, T. Y, Partin, A. W, Walsh, P. C, & Epstein, J. I. Prognostic significance of gleason score 3+ 4 versus gleason score 4+ 3 tumor at radical prostatectomy. *Urology*, **56**(5), 823–827 (2000). 10.1016/s0090-4295(00)00753-6 11068310

[5] Chandran, U. R, Ma, Changqing, Dhir, R., Bisceglia, M., Lyons-Weiler, M., Liang, W., Michalopoulos, G., Becich, M., & Monzon, F. A. Gene expression profiles of prostate cancer reveal involvement of multiple molecular pathways in the metastatic process. *BMC cancer*, **7**(1), 1–21 (2007). 10.1186/1471-2407-7-64 PMC1865555 17430594

[6] Cheerla A, Gevaert O (2019). Deep learning with multimodal representation for pancancer prognosis prediction. Bioinformatics.

[7] Chen, J. W, Dhahbi, J. Lung adenocarcinoma and lung squamous cell carcinoma cancer classification, biomarker identification, and gene expression analysis using overlapping feature selection methods. *Sci. Rep.*, **11**(1), 1–15 (2021). 10.1038/s41598-021-92725-8 PMC8233431 34172784

[8] Chitale, D., Gong, Y., Taylor, B. S., Broderick, S., Brennan, C., Somwar, R., Golas, B., Wang, Lu, M. N., & Szoke, J. et al. An integrated genomic analysis of lung cancer reveals loss of dusp4 in egfr-mutant tumors. *Oncogene*, **28**(31), 2773–2783 (2009). 10.1038/onc.2009.135 PMC2722688 19525976

[9] Dai X, Cheng H, Bai Z, Li J (2017). Breast cancer cell line classification and its relevance with breast tumor subtyping. J. Cancer.

[10] Duan, K., Sathiya Keerthi, S, & Poo, Aun N. Evaluation of simple performance measures for tuning svm hyperparameters. *Neurocomputing*, **51**, 41–59 (2003).

[11] Duroux D, Van Steen K (2023). netanova: novel graph clustering technique with significance assessment via hierarchical anova. Briefings in Bioinform.

[12] Egevad, Lars, Granfors, T, Karlberg, L, Bergh, A, & Stattin, Per. Prognostic value of the gleason score in prostate cancer. *BJU international*, **89**(6), 538–542 (2002). 10.1046/j.1464-410x.2002.02669.x 11942960

[13] Ferwerda J, Hainmueller J, Hazlett CJ (2017). Kernel-based regularized least squares in R (KRLS) and Stata (krls). J. Stat. Softw..

[14] Frandsen, J., Orton, A., Shrieve, D., & Tward, J. Risk of death from prostate cancer with and without definitive local therapy when gleason pattern 5 is present: a surveillance, epidemiology, and end results analysis. *Cureus*, **9**(7), (2017). 10.7759/cureus.1453 PMC5590810 28929037

[15] Garufi A, Traversi G, Cirone M, D’Orazi G (2019). Hipk2 role in the tumor-host interaction: impact on fibroblasts transdifferentiation caf-like. IUBMB Life.

[16] Gregorich M, Melograna F, Sunqvist M, Michiels S, Van Steen K, Heinze G (2022). Individual-specific networks for prediction modelling-a scoping review of methods. BMC Med. Res. Methodol..

[17] Guebila, M. B., Wang, T., Lopes-Ramos, C. M., Fanfani, V., Weighill, D., Burkholz, R., Schlauch, D., Paulson, J. N, Altenbuchinger, M., & Sonawane, A.*et al*. The network zoo: a multilingual package for the inference and analysis of biological networks. *bioRxiv*, (2022). 10.1186/s13059-023-02877-1 PMC9999668 36894939

[18] Harrell, F. E, Jr. with contributions from Charles Dupont, and many others. *Hmisc: Harrell Miscellaneous*, (2021). R package version 4.5-0.

[19] He, K., Zhang, X., Ren, S., & Sun, J. Deep residual learning for image recognition. *CoRR*, abs/1512.03385, (2015).

[20] Hengel H, Keimer R, Deigendesch W, Rieß A, Marzouqa H, Zaidan J, Bauer P, Schöls L (2018). Gpt2 mutations cause developmental encephalopathy with microcephaly and features of complicated hereditary spastic paraplegia. Clin. Genet..

[21] Holzinger A, Malle B, Saranti A, Pfeifer B (2021). Towards multi-modal causability with graph neural networks enabling information fusion for explainable ai. Inform. Fusion.

[22] Hu, J., Li, X., Coleman, K., Schroeder, A., Ma, N., Irwin, D. J., Lee, E. B, Shinohara, R. T., & Li, M. S. Integrating gene expression, spatial location and histology to identify spatial domains and spatially variable genes by graph convolutional network. *Nature methods*, **18**(11), 1342–1351 (2021). 10.1038/s41592-021-01255-8 34711970

[23] Huang, H.-C., Chuang, Y.-Y., & Chen, C.-S. Affinity aggregation for spectral clustering. In *2012 IEEE Conference on Computer Vision and Pattern Recognition*, pages 773–780. IEEE, (2012).

[24] Ilse, M., Tomczak, J., Welling, M. Attention-based deep multiple instance learning. In Jennifer Dy and Andreas Krause, editors, *Proceedings of the 35th International Conference on Machine Learning*, volume 80 of *Proceedings of Machine Learning Research*, pages 2127–2136. PMLR, 10–15 (Jul 2018).

[25] Karatzoglou A, Smola A, Hornik K, Zeileis A (2004). kernlab-an s4 package for kernel methods in r. J. Stat. Softw..

[26] Karim, Md R., Wicaksono, G., Costa, I. G., Decker, S., Beyan, O. Prognostically relevant subtypes and survival prediction for breast cancer based on multimodal genomics data. *IEEE Access*, **7**, 133850–133864 (2019).

[27] Kaymakcalan, H., Yarman, Y., Goc, N., Toy, F., Meral, C., Ercan-Sencicek, A. G., & Gunel, M. Novel compound heterozygous mutations in gpt2 linked to microcephaly, and intellectual developmental disability with or without spastic paraplegia. *Am. J. Med. Gen. Part A*, **176**(2), 421–425 (2018). 10.1002/ajmg.a.38558 29226631

[28] Khan, Md T., Prajapati, B., Lakhina, S., Sharma, M., Prajapati, S., Chosdol, K., & Sinha, S. Identification of gender-specific molecular differences in glioblastoma (gbm) and low-grade glioma (lgg) by the analysis of large transcriptomic and epigenomic datasets. *Front. Oncol.*, 11, (2021). 10.3389/fonc.2021.699594 PMC8491982 34621669

[29] Koh, H.W.L., Fermin, D., Vogel, C., Choi, K. P., Ewing, R. M., & Choi, H. iomicspass: network-based integration of multiomics data for predictive subnetwork discovery. *NPJ systems biology and applications*, **5**(1), 1–10 (2019). 10.1038/s41540-019-0099-y PMC6616462 31312515

[30] Kuijjer, M. L, Hsieh, P.-H., Quackenbush, J., & Glass, K. lionessr: single sample network inference in r. *BMC cancer*, **19**(1), 1–6 (2019). 10.1186/s12885-019-6235-7 PMC6815019 31653243

[31] Kuijjer, M. L. *lionessR: Modeling networks for individual samples using LIONESS*, (2022). R package version 1.0.

[32] Marieke Lydia Kuijjer (2019). Matthew George Tung, GuoCheng Yuan, John Quackenbush, and Kimberly Glass. Estimating sample-specific regulatory networks. Iscience.

[33] Lavery, Hugh J, & Droller, Michael J. Do gleason patterns 3 and 4 prostate cancer represent separate disease states? *J. Urol.*, **188**(5), 1667–1675 (2012). 10.1016/j.juro.2012.07.055 22998919

[34] Liberzon A, Birger C, Thorvaldsdóttir H, Ghandi M, Mesirov JP, Tamayo P (2015). The molecular signatures database hallmark gene set collection. Cell Syst..

[35] Liu, P., Ramachandran, S., Seyed, M. A., Scharer, C. D., Laycock, N., Dalton, W. B., Williams, H., Karanam, S., Datta, M. W, Jaye, D. L *et al*. Sex-determining region y box 4 is a transforming oncogene in human prostate cancer cells. *Cancer Res.*, **66**(8), 4011–4019 (2006). 10.1158/0008-5472.CAN-05-3055 16618720

[36] Liu T, Huang J, Liao T, Pu R, Liu S, Peng Y (2022). A hybrid deep learning model for predicting molecular subtypes of human breast cancer using multimodal data. Irbm.

[37] Liu X, Wang Y, Ji H, Aihara K, Chen L (2016). Personalized characterization of diseases using sample-specific networks. Nucleic Acids Res..

[38] McInnes, L., Healy, J., & Melville, J. Umap: Uniform manifold approximation and projection for dimension reduction. arXiv preprint arXiv:1802.03426, (2018).

[39] Menche, Jörg, G., Emre, S., Amitabh, B., Patrick J, L., Matthew J., Baribaud, Frédéric, D. R, & Barabási, A. Integrating personalized gene expression profiles into predictive disease-associated gene pools. *NPJ Syst. Biol. Appl.*, **3**(1), 1–10 (2017). 10.1038/s41540-017-0009-0 PMC5445628 28649437

[40] Mobadersany, P., Yousefi, S., Amgad, M., Gutman, D. A, Barnholtz-Sloan, J. S, Vega, J. E., Velázquez, B., Daniel, J., & Cooper, L.A.D. Predicting cancer outcomes from histology and genomics using convolutional networks. *Proceedings of the National Academy of Sciences*, **115**(13), E2970–E2979 (2018). 10.1073/pnas.1717139115 PMC5879673 29531073

[41] Ouyang, Q., Nakayama, T., Baytas, O., Davidson, S. M, Yang, C., Schmidt, M., Lizarraga, S. B, Mishra, S., Malak, EI-Quessny, N. S.*et al.* Mutations in mitochondrial enzyme gpt2 cause metabolic dysfunction and neurological disease with developmental and progressive features. *Proceedings of the National Academy of Sciences*, **113**(38), E5598–E5607 (2016). 10.1073/pnas.1609221113 PMC5035873 27601654

[42] Ritchie, M. E., Phipson, B., Wu, D., Hu, Y., Law, C. W., Shi, W., & Smyth, G. K. limma powers differential expression analyses for rna-sequencing and microarray studies. *Nucleic acids Res.*, **43**(7), e47–e47 (2015). 10.1093/nar/gkv007 PMC4402510 25605792

[43] Schmauch B, Romagnoni A, Pronier E, Saillard C, Maillé P, Calderaro J, Kamoun A, Sefta M, Toldo S, Zaslavskiy M (2020). A deep learning model to predict rna-seq expression of tumours from whole slide images. Nat. Commun..

[44] Schneider, L., Laiouar-Pedari, S., Kuntz, S., Krieghoff-Henning, E., Hekler, A., Kather, J. N., Gaiser, T., Fröhling, S,, & Brinker, T. J. Integration of deep learning-based image analysis and genomic data in cancer pathology: a systematic review. *Eur J Cancer*, **160**, 80–91 (2022). 10.1016/j.ejca.2021.10.007 34810047

[45] Sergushichev, A. An algorithm for fast preranked gene set enrichment analysis using cumulative statistic calculation. *bioRxiv*, (2016).

[46] Shedden, K., Taylor, J.M.G., Enkemann, S. A, Tsao, M. S, Yeatman, T. J., Gerald, W. L., Eschrich, S., Jurisica, I., Venkatraman, S. E, Meyerson, M. *et al.* Gene expression-based survival prediction in lung adenocarcinoma: a multi-site, blinded validation study: Director’s challenge consortium for the molecular classification of lung adenocarcinoma. *Nature medicine*, **14**(8), 822 (2008). 10.1038/nm.1790 PMC2667337 18641660

[47] Shen, R, Mo, Q., Schultz, N., Seshan, V. E., Olshen, A. B., Huse, J., Ladanyi, M, & Sander, C. Integrative subtype discovery in glioblastoma using icluster. *PloS one*, **7**(4), e35236 (2012). 10.1371/journal.pone.0035236 PMC3335101 22539962

[48] Shen, R., Olshen, A. B, & Ladanyi, M. Integrative clustering of multiple genomic data types using a joint latent variable model with application to breast and lung cancer subtype analysis. *Bioinformatics*, **25**(22), 2906–2912 (2009). 10.1093/bioinformatics/btp543 PMC2800366 19759197

[49] Speicher, N. K., Pfeifer, N. Integrating different data types by regularized unsupervised multiple kernel learning with application to cancer subtype discovery. *Bioinformatics*, **31**(12), i268–i275 (2015). 10.1093/bioinformatics/btv244 PMC4765854 26072491

[50] Subramanian A, Tamayo P, Mootha VK, Mukherjee S, Ebert BL, Gillette MA, Paulovich A, Pomeroy SL, Golub TR, Lander ES, Mesirov JP (2005). Gene set enrichment analysis: a knowledge-based approach for interpreting genome-wide expression profiles. Proc. Natl. Acad. Sci..

[51] Tran, D., Nguyen, H., Le, U., Bebis, G., Luu, H. N., & Nguyen, T. A novel method for cancer subtyping and risk prediction using consensus factor analysis. *Front. Oncol.*, page 1052 (2020). 10.3389/fonc.2020.01052 PMC7344292 32714868

[52] Wang, B., Mezlini, A., Demir, F., Fiume, M., Tu, Z., Brudno, M., Haibe-Kains, B., & Goldenberg, A. *SNFtool: Similarity Network Fusion*, (2021). R package version 2.3.1. 10.1038/nmeth.2810 24464287

[53] Wang, B., Mezlini, A. M., Demir, F., Fiume, M., Tu, Z., Brudno, M., Haibe-Kains, B., & Goldenberg, A. Similarity network fusion for aggregating data types on a genomic scale. *Nature methods*, **11**(3), 333–337 (2014). 10.1038/nmeth.2810 24464287

[54] Weinstein, J. N., Collisson, E. A., Mills, G. B., Shaw, K. R., Ozenberger, B. A., Ellrott, K., Shmulevich, I., Sander, C., & Stuart, J. M. The cancer genome atlas pan-cancer analysis project. *Nature Gen.*, **45**(10), 1113–1120 (2013). 10.1038/ng.2764 PMC3919969 24071849

[55] Zagars, G. K., Ayala, A. G., von Eschenbach, A. C., & Pollack, A. The prognostic importance of gleason grade in prostatic adenocarcinoma: a long-term follow-up study of 648 patients treated with radiation therapy. *Int. J. Radiation Oncol. Biol. Phys.*, **31**(2), 237–245 (1995). 10.1016/0360-3016(94)00323-D 7836075

[56] Zhang Z, Wen P, Li F, Yao C, Wang T, Liang B, Yang Q, Ma L, He L (2018). Hipk2 inhibits cell metastasis and improves chemosensitivity in esophageal squamous cell carcinoma. Exp. Ther. Med..

[57] Zhong T, Mengyun W, Ma S (2019). Examination of independent prognostic power of gene expressions and histopathological imaging features in cancer. Cancers.

