# Supplementary material for: Graph-based multi-modality integration for prediction of cancer subtype and severity
Source: Sci Rep. 2023 Nov 10;13:19653. doi: 10.1038/s41598-023-46392-6 (PMC10638406; doi:10.1038/s41598-023-46392-6)
Supplement: Supplementary file 1 — Supplementary Information. [file 41598_2023_46392_MOESM1_ESM.pdf]

# Graph-based multi-modality integration for prediction of cancer subtype and severity

Diane Duroux[1,5*], Christian Wohlfart[2], Kristel Van Steen[1,3], Antoaneta Vladimirova[4], Michael King[2]

[1]BIO3 - Systems Genetics, GIGA-R Medical Genomics, University of Liège, 4000 Liège, Belgium
[2]Roche Diagnostics GmbH, Penzberg, Germany.
[3]BIO3 - Systems Medicine, Department of Human Genetics, KU Leuven, 3000 Leuven, Belgium.
[4]Roche Information Solutions, Roche Diagnostics Corporation, Santa Clara, California, United States of America
[5]Post-Doctoral Fellow, ETH AI center
*Corresponding authors: diane.duroux@ai.ethz.ch

## SUPPLEMENTARY

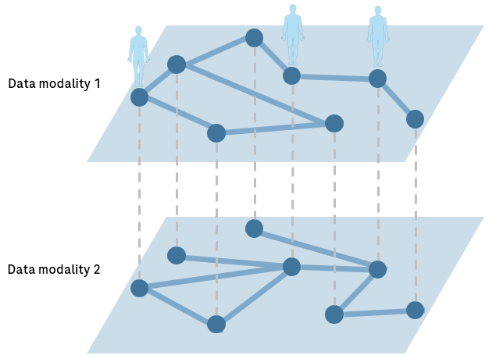


(a)

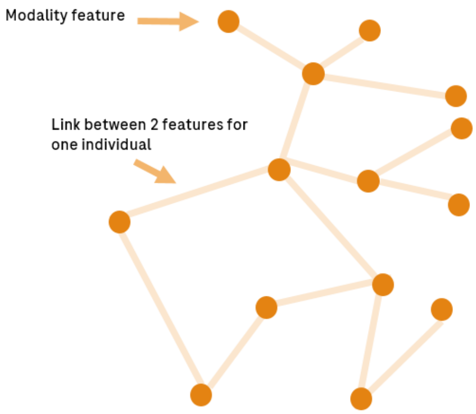


(b)

**Fig. 1:** **(a)** Multi-modality fusion from Person-to-Person networks. Nodes are individuals and edges show how close 2 individuals are. **(b)** Individual network. Nodes and/or edges are individual-specific.

| | |
|---|---|
| ACC | Adrenocortical carcinoma |
| BLCA | Bladder Urothelial Carcinoma |
| LGG | Brain Lower Grade Glioma |
| BRCA | Breast invasive carcinoma |
| CESC | Cervical squamous cell carcinoma and endocervical adenocarcinoma |
| CHOL | Cholangiocarcinoma |
| COAD | Colon adenocarcinoma |
| ESCA | Esophageal carcinoma |
| GBM | Glioblastoma multiforme |
| HNSC | Head and Neck squamous cell carcinoma |
| KIRP | Kidney renal papillary cell carcinoma |
| LIHC | Liver hepatocellular carcinoma |
| LUAD | Lung adenocarcinoma |
| LUSC | Lung squamous cell carcinoma |
| DLBC | Lymphoid Neoplasm Diffuse Large B-cell Lymphoma |
| MESO | Mesothelioma |
| OV | Ovarian serous cystadenocarcinoma |
| PAAD | Pancreatic adenocarcinoma |
| PCPG | Pheochromocytoma and Paraganglioma |
| PRAD | Prostate adenocarcinoma |
| READ | Rectum adenocarcinoma |
| SARC | Sarcoma |
| SKCM | Skin Cutaneous Melanoma |
| STAD | Stomach adenocarcinoma |
| TGCT | Testicular Germ Cell Tumors |
| THYM | Thymoma |
| THCA | Thyroid carcinoma |
| UCS | Uterine Carcinosarcoma |
| UCEC | Uterine Corpus Endometrial Carcinoma |
| UVM | Uveal Melanoma |

**Table 1:** Cancer types used for histopathology data feature extraction.

| | Prostate | | | Brain | | | Lung | | |
|---|---|---|---|---|---|---|---|---|---|
| | RNASeq | Histo. | Fusion | RNASeq | Histo. | Fusion | RNASeq | Histo. | Fusion |
| Pen. log. reg. | 77/74 | 78/71 | 83/83 | 96/99 | 93/98 | 97/99 | 94/86 | 92/85 | 97/64 |
| Tree | 56/71 | 54/60 | 46/69 | 88/97 | 67/92 | 82/95 | 97/95 | 83/71 | 97/95 |
| Random forest | 56/73 | 66/74 | 67/78 | 96/99 | 85/96 | 99/99 | 97/94 | 92/84 | 97/95 |
| Naive Bayes | 69/72 | 78/71 | 82/82 | 88/96 | 71/89 | 94/98 | 95/91 | 89/77 | 96/93 |
| AdaBoost | 62/76 | 71/78 | 75/81 | 96/99 | 89/97 | 100/100 | 97/95 | 91/83 | 97/95 |
| Neural network | 51/56 | 67/71 | 34/61 | 58/91 | 91/96 | 54/89 | 86/67 | 93/87 | 83/67 |
| Graph approach | 66/76 | 69/72 | 79/79 | 99/100 | 90/97 | 100/100 | 98/96 | 96/92 | 98/96 |

**Table 2:** Class-wise F1 scores (%), ie $F1_{group1}/F1_{group2}$, for the different inputs (RNASeq data only, histopathology images only or fusion of the two modalities) and algorithms evaluated.

### Relevance of differentiating Gleason score 3 versus 4

The relatively benign nature of homogeneous, low-volume Gleason 3 tumors stands in contrast to the progressive risk of biochemical recurrence and prostate cancer-specific mortality associated with increasing quantities of Gleason 4 components [5]. Notably, these differences underscore the existence of distinct cancer diatheses, each demanding tailored approaches. Furthermore, tumors with Gleason score 3+4 or Gleason score 4+3 are characterized by significant heterogeneity in their biological behavior [2]. The prognosis for patients with Gleason scores 3+4 and 4+3 tumors at radical prostatectomy exhibits notable differences.

### Model comparison

We compared our graph-based approach and its variants to several classification methods applied to the raw features. Data were pre-processed as in the graph approach, i.e. the same variables were selected (Section 3.1). For the penalized logistic regression, we used function *cv.glmnet* from the package *glmnet* [3] with options $\text{alpha} = 1, \text{lambda} = \text{NULL}$. For the random forest, we applied function *randomForest* from the package *randomForest* [6] with option ntree = 500. For AdaBoost, we used the function *boosting* from the package *adabag* [1] with option boos = $TRUE$, and mfinal = 50. For the classification tree, we applied the function *rpart* from the package *rpart*[8] with the default options. For the naive Bayes approach, we used the function *naiveBayes* from the package *e1071* [7] with the default options. Finally, we applied a neural network. In particular, we have used the *neuralnet* function from the *neuralnet* R package [4]. The neural network consists of two hidden layers and we set the number of neurons per layer $\sqrt{\#\text{ nodes in the previous layer} \times \#\text{ nodes in the output layer}}$. The parameter linear.output = FALSE and default options were used.

### Effect of data imbalance

While the workflow doesn't include a preprocessing step directly on the input data, such as under or over-sampling, it incorporates strategies aimed at alleviating the impact of data imbalance. Firstly, the dataset-specific feature selection step includes the use of appropriate evaluation metrics designed for imbalanced data. Indeed, to determine the optimal feature selection thresholds, we conducted a stratified 5-fold cross-validation within the training set, choosing parameters that yielded the highest average macro F1 score. This approach ensures that the feature selection process focuses on maintaining a balance between classes. Furthermore, when tuning hyperparameters for the Support Vector Machine, we rely on cross-validation evaluated using the macro F1 score as well. This fine-tunes our models to perform well in imbalanced settings. An alternative option would have been to employ a class-weighted SVM, which addresses unbalanced data by assigning higher misclassification penalties to training instances of the minority class.

To provide a more comprehensive evaluation of the models' performance and assess potential bias towards specific classes, the class-wise F1 scores are summarized in Table 2. With the graph-based approach, the difference between the two F1 scores stands at 3.3% on average. In two of the nine analyses (columns), the F1 score is even equal between the two classes. The largest discrepancy (11%), is observed in the context of prostate cancer classification based on RNASeq data. Overall, these findings indicate that there is no substantial disparity between the F1 scores achieved in the two groups, and suggests that the approach adeptly handles data imbalance.

| | Nodes | | | Edges | | Nodes and edges | |
|---|---|---|---|---|---|---|---|
| | Spearman | Euclidean | Gaussian kernel | Node product | LIONESS | Spearman + Node product | Spearman + LIONESS |
| RNAseq data only | x | x | x | x | x | x | x |
| Histopathology images only | x | x | x | x | x | x | x |
| Early fusion | x | x | x | x | x | x | x |
| Intermediate fusion via average | x | x | x | x | x | x | x |
| Intermediate fusion via SNF | x | x | x | x | | | |
| Late fusion | | | | | | x | x |

**Fig. 2:** Overview of the workflow variations evaluated. The rows describe the data fusion methods, and columns show the information used to build the Person-to-Person Network.

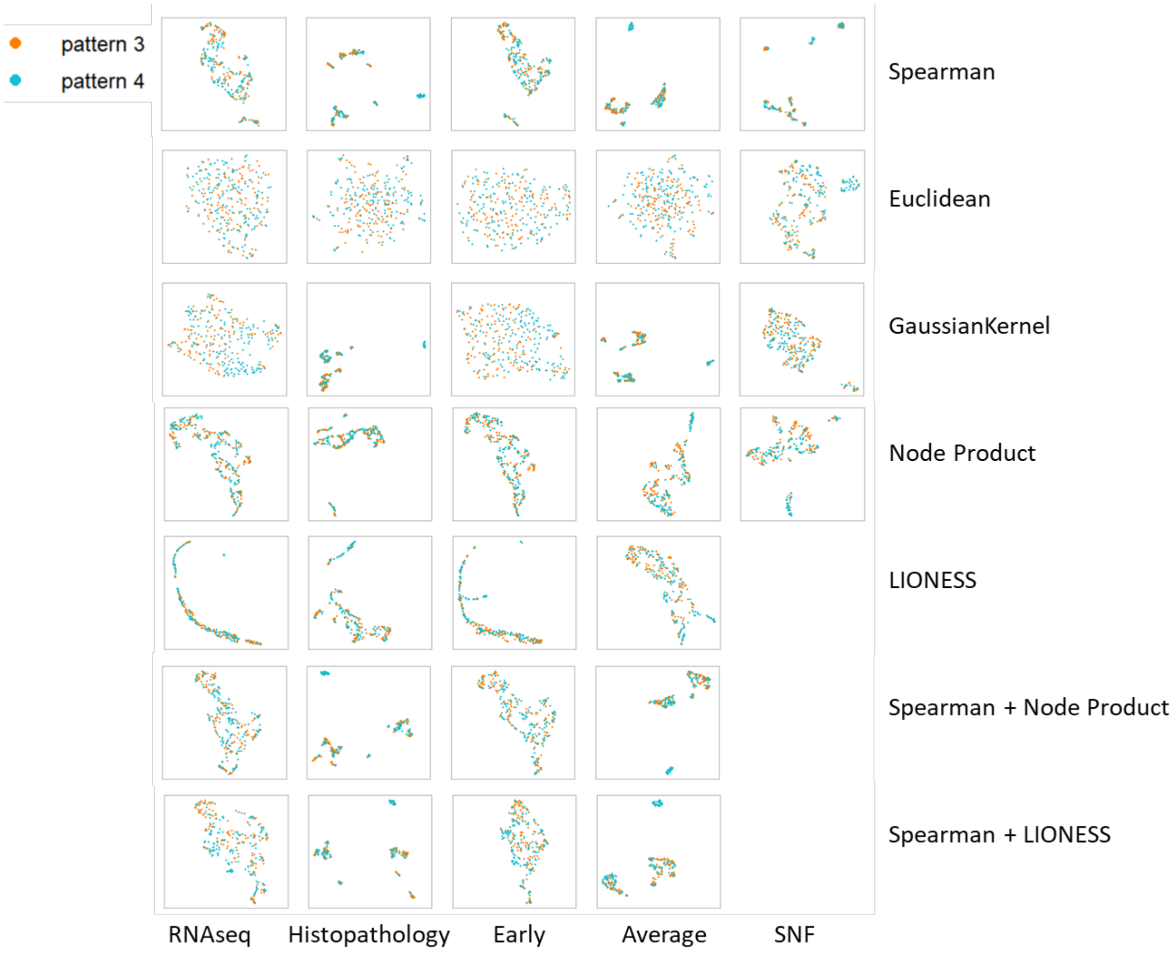


**Fig. 3:** Visualization of the PPNs used as input for SVM-based prostate cancer subtype prediction, created using UMAP. The three first rows refer to approaches based on the nodes of the individual networks. Rows 4 and 5 use the edge weights of the individual networks. Rows 6 and 7 combine individual nodes and edges. The two first columns focus on a single data modality. Columns 3 to 5 refer to data integration.

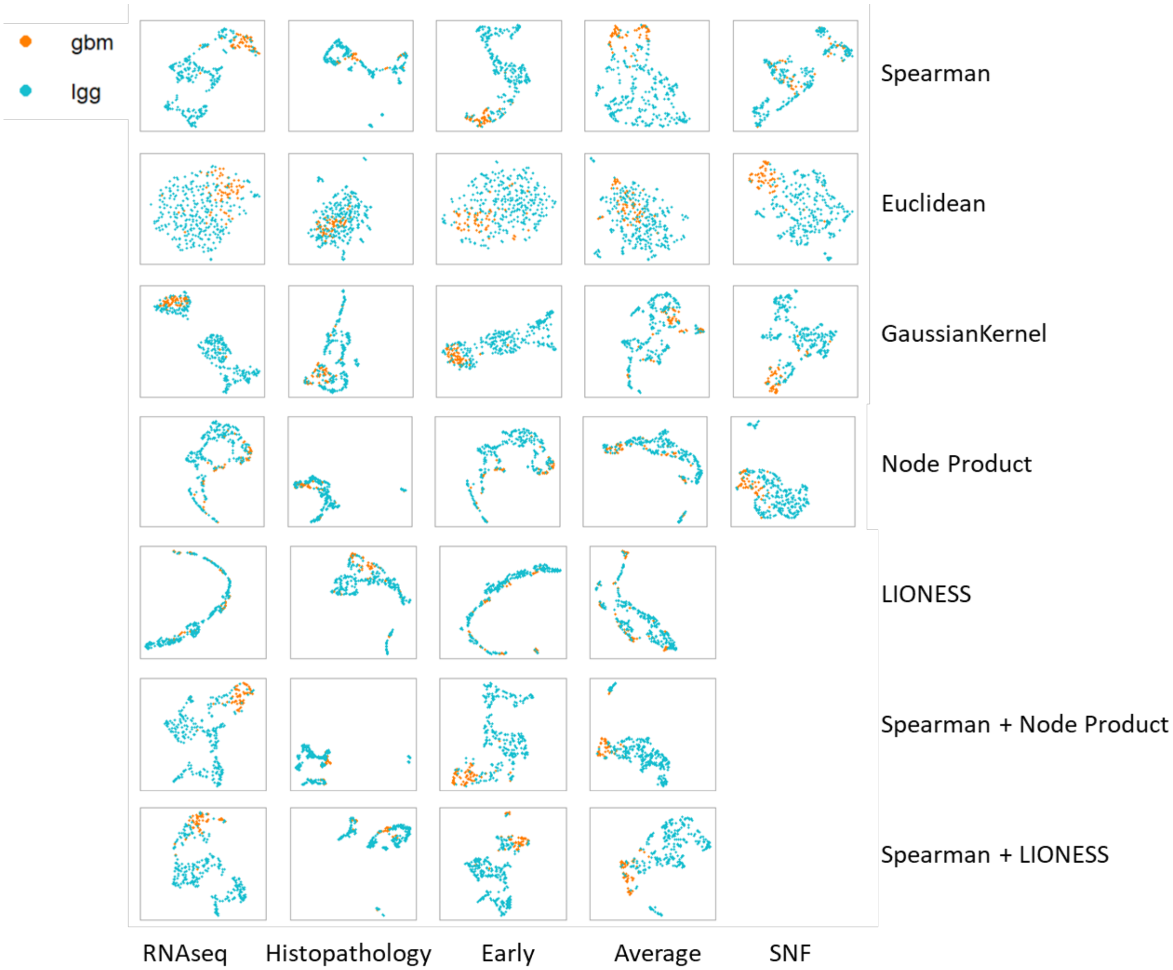


**Fig. 4:** Visualization of the PPNs used as input for SVM-based prostate brain subtype prediction, created using UMAP. The three first rows refer to approaches based on the nodes of the individual networks. Rows 4 and 5 use the edge weights of the individual networks. Rows 6 and 7 combine individual nodes and edges. The two first columns focus on a single data modality. Columns 3 to 5 refer to data integration.

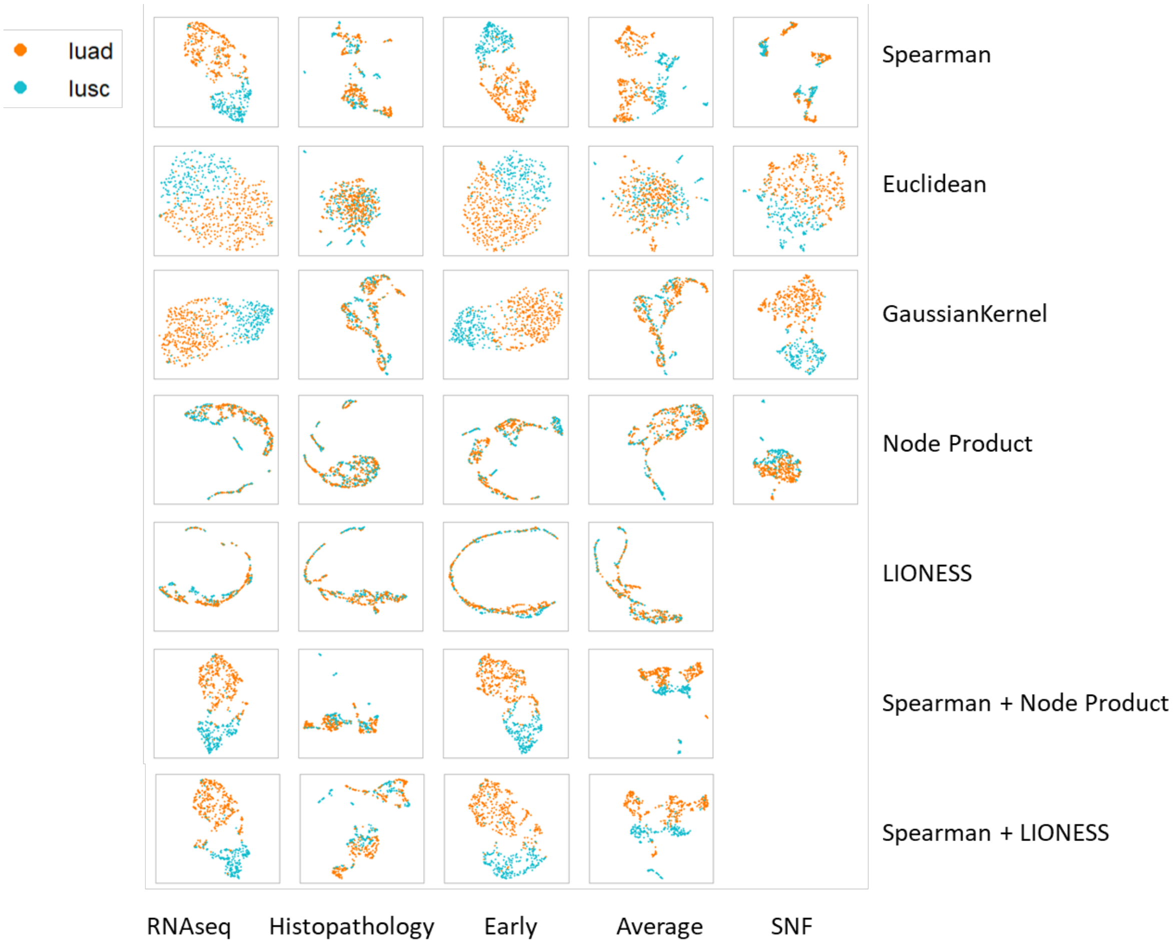


**Fig. 5:** Visualization of the PPNs used as input for SVM-based lung cancer subtype prediction, created using UMAP. The three first rows refer to approaches based on the nodes of the individual networks. Rows 4 and 5 use the edge weights of the individual networks. Rows 6 and 7 combine individual nodes and edges. The two first columns focus on a single data modality. Columns 3 to 5 refer to data integration.

(a)

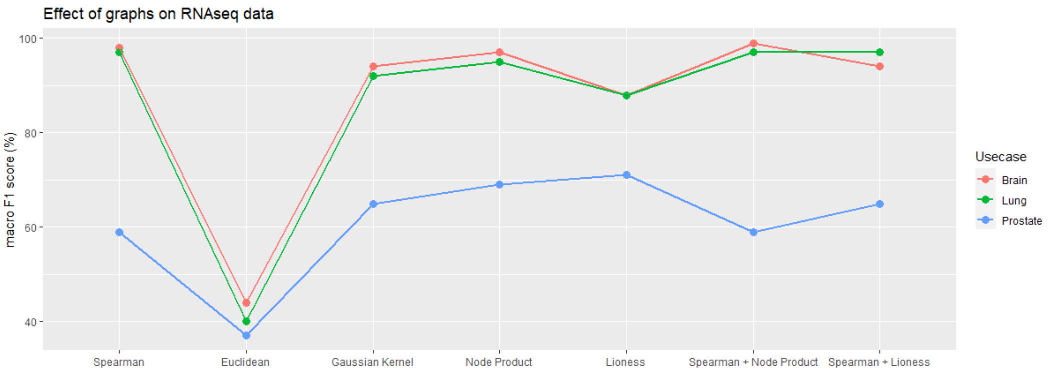


(b)

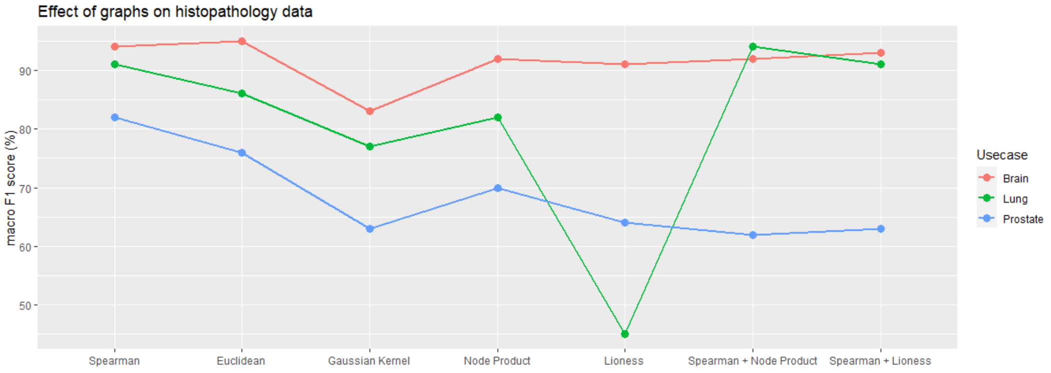


**Fig. 6:** Overview of the macro F1 scores (%) for multiple data transformation on RNAseq data **(a)** (resp. histopathology data **(b)**), described in Section 2.2. No graph information is used when inferring the similarity matrix using the Spearman correlation, the euclidean distance or the Gaussian kernel. Only graph information are studied when similarities are computed from individual graphs built with the *Node Product* or the *LIONESS* algorithm. Both *raw data* and graph information are investigated when a combination of the similarity matrices obtained with Spearman correlation and the *Node Product* of, with Spearman correlation and Lioness.

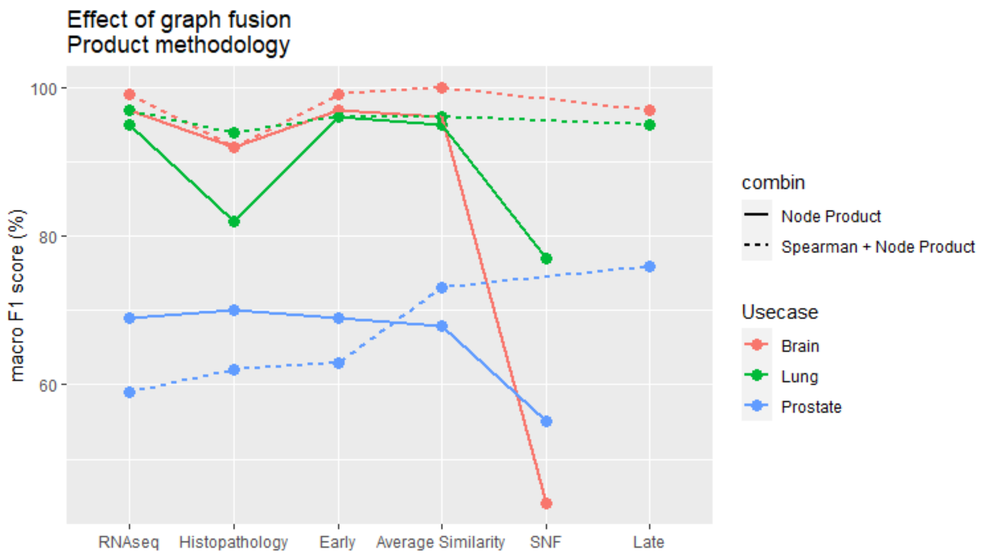


(a)

(b)

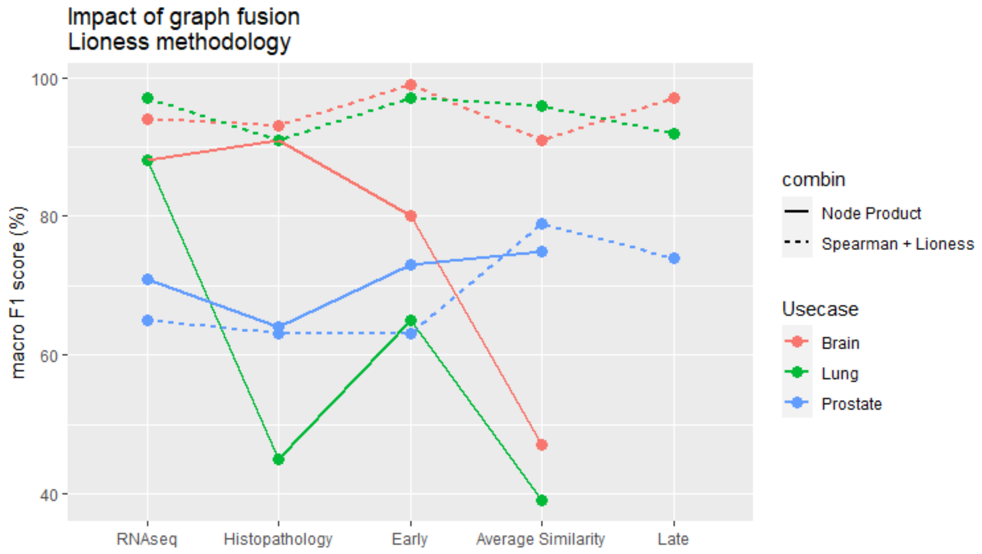


**Fig. 7:** Overview of the macro F1 scores (%) for fusion of graphs at different level. Graphs are constructed via the *Node Product* methodology **(a)** and the Lioness approach **(b)**, described in Section 2.2. Only one data type is used for values *RNAseq* and *Histopathology*. Data types are combined at early stage via the concatenation of the two databases (*early*), at intermediate stage via average of the similarity matrices (*average*) or SNF procedure (*SNF*) and at late stage via the majority vote (*late*). Note that the late integration is only performed when the combination of *raw data* and graph data are used so that the majority vote is applied on more that 2 outcomes (Section 2.3).

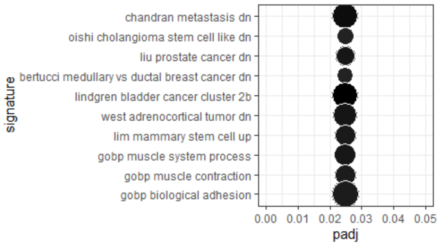


(a)

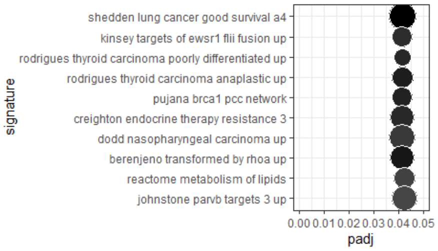


(b)

**Fig. 8:** **(a)** (resp. **(b)**) display the top 10 enriched gene sets from the largest component obtained from LIMMA analysis with features selected as describes in Section 2 for the prostate cancer (resp. Lung cancer). No enriched gene set is detected in the Brain cancer use case. The size of a pathway represents the number of genes in this pathway after removing genes not present in the largest component.

## References


[1] Esteban Alfaro, Matías Gámez, and Noelia García. adabag: An R package for classification with boosting and bagging. *Journal of Statistical Software*, 54(2):1–35, 2013.

[2] Theresa Y Chan, Alan W Partin, Patrick C Walsh, and Jonathan I Epstein. Prognostic significance of gleason score 3+ 4 versus gleason score 4+ 3 tumor at radical prostatectomy. *Urology*, 56(5):823–827, 2000.

[3] Jerome Friedman, Trevor Hastie, and Rob Tibshirani. Regularization paths for generalized linear models via coordinate descent. *Journal of statistical software*, 33(1):1, 2010.

[4] Frauke Günther and Stefan Fritsch. Neuralnet: training of neural networks. *R J.*, 2(1):30, 2010.

[5] Hugh J Lavery and Michael J Droller. Do gleason patterns 3 and 4 prostate cancer represent separate disease states? *The Journal of urology*, 188(5):1667–1675, 2012.

[6] Andy Liaw and Matthew Wiener. Classification and regression by randomforest. *R News*, 2(3):18–22, 2002.

[7] David Meyer, Evgenia Dimitriadou, Kurt Hornik, Andreas Weingessel, and Friedrich Leisch. *e1071: Misc Functions of the Department of Statistics, Probability Theory Group (Formerly: E1071), TU Wien*, 2022. R package version 1.7-11.

[8] Terry Therneau and Beth Atkinson. *rpart: Recursive Partitioning and Regression Trees*, 2019. R package version 4.1-15.
